# Anode‐Free Sodium Metal Batteries: From Materials Design to System‐Level Integration

**DOI:** 10.1002/advs.77874

**Published:** 2026-09-30

**Authors:** Hamid Hussain, Shazaib Ali, Muhammad Ali, Samia Aman, Moazzam Ali, Ufra Naseer, Sajid Farooq, Asif Mahmood, Sailing He, Yinzhu Jiang, Muhammad Yousaf

**Affiliations:** ^1^ School of Materials Science and Engineering Zhejiang University Hangzhou China; ^2^ Future Science Research Institute ZJU‐Hangzhou Global Scientific and Technological Innovation Center Zhejiang University Hangzhou China; ^3^ School of Chemical and Biological Engineering Zhejiang University Hangzhou China; ^4^ School of Chemistry Zhejiang University Hangzhou China; ^5^ National Engineering Research Center for Optical Instruments College of Optical Science and Engineering Zhejiang University Hangzhou China; ^6^ College of Mathematical Medicine Zhejiang Normal University Jinhua China; ^7^ Institute of Sustainable Industries and Liveable Cities Victoria University Melbourne Victoria Australia; ^8^ Department of Electromagnetic Engineering School of Electrical Engineering KTH Royal Institute of Technology Stockholm Sweden

**Keywords:** anode‐free sodium metal batteries, interfacial engineering, pouch‐cell scaling, predictive design, sodium‐inventory‐limited systems, system‐level integration

## Abstract

Anode‐free sodium metal batteries (AFSMBs) offer a lithium‐independent route to high‐energy, low‐cost storage, but their deployment is constrained by limited sodium inventory and interfacial instability. Without excess sodium, small inefficiencies in nucleation, interphase formation, and cross‐electrode reactions accumulate into irreversible capacity loss, and advances in materials design frequently fail to survive practical conditions. Here we establish a mechanism‐informed framework that resolves the sodium inventory into reversible, dead, and chemically bound fractions, each tied to a measurable quantity, and apply it across current collectors, electrolytes, cathodes, separators, and solid‐state architectures. Because Coulombic efficiency reports only the sum of two loss channels that scale differently, we derive the efficiency required for practical operation rather than assuming it: 500 cycles at N/P = 0 demands an average above 99.95%. Screened against lean‐electrolyte and high‐areal‐capacity criteria, few reported systems approach this threshold, and most omit the electrolyte loading at which their efficiency is obtained. We identify operando diagnostics and quantitative morphological descriptors as the missing tools for predictive design. AFSMBs are therefore integration‐limited rather than materials‐limited, and progress depends on translating interfacial control into manufacturable cell architectures.

## Graphical abstract

Anode‐free sodium metal batteries hold no sodium reserve, so every inefficiency becomes permanent loss. Resolving the inventory into reversible, dead, and chemically bound fractions exposes what Coulombic efficiency hides. Practical 500‐cycle operation requires above 99.95%, a threshold few systems meet under lean electrolyte and high areal capacity, revealing an integration‐limited field.

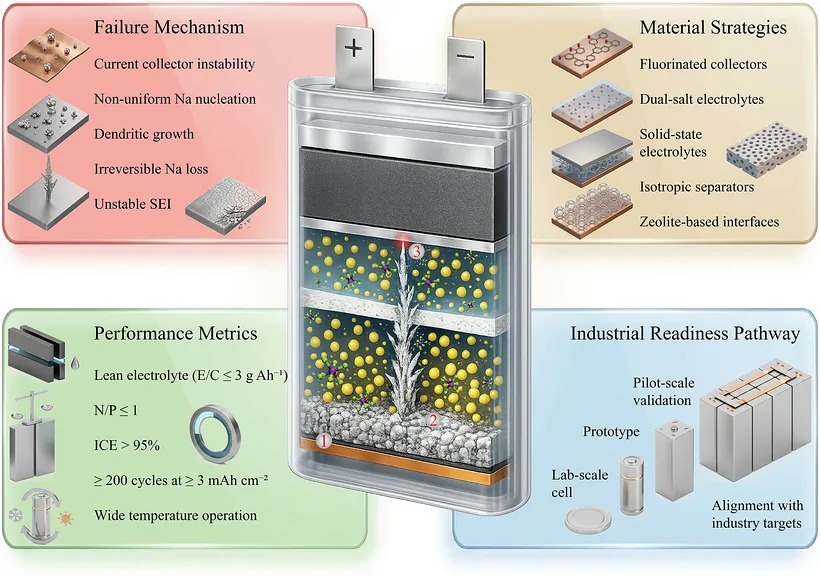

## 1 Introduction

Electrochemical systems that operate without excess active‐metal reservoirs exhibit fundamentally different failure behavior, in which cumulative inventory loss, rather than fully reversible processes, governs performance. Such constraints are increasingly relevant in the context of next‐generation energy storage. The rapid expansion of electrified transport and renewable energy storage is increasing demand for lithium‐ion batteries, exposing challenges in resource availability and cost stability [1, 2, 3]. These limitations have renewed interest in sodium‐based systems, which rely on earth‐abundant elements and offer a more sustainable and geographically diversified alternative for large‐scale energy storage [4, 5]. Recent advances in sodium‐ion batteries have enabled prototype cells that approach commercial benchmarks, using layered oxide cathodes and hard‐carbon anodes [6, 7]. However, their energy density remains limited (≈120 Wh kg^−1^) by the capacity of hard‐carbon anodes and Na‐rich cathodes [8, 9]. Anode‐free sodium metal batteries (AFSMBs) have therefore emerged as an alternative architecture, in which metallic sodium is reversibly plated onto a bare current collector during operation [10, 11].

Despite this conceptual simplicity, AFSMBs are governed by complex interfacial processes. Sodium exhibits weaker substrate binding and a larger ionic radius than lithium, together with distinct solvation behavior, leading to higher nucleation overpotentials, mechanically soft deposits, and unstable interphases [12, 13, 14, 15]. These effects promote heterogeneous growth, repeated fracture of the solid–electrolyte interphase (SEI), and continuous sodium consumption, which are particularly detrimental in the absence of a metallic reservoir for compensation [16, 17, 18, 19].

Although significant progress has been achieved in electrolyte, interface, and structural design, advances remain largely focused on individual components and often fail to translate to practical conditions, where lean electrolyte operation, low N/P ratios, and cell‐level constraints govern stability. This indicates that AFSMB performance is determined by system‐level integration rather than isolated materials optimization.

Here, we present a mechanism‐informed framework that links sodium deposition behavior, interphase evolution, and failure propagation across cell components (Figure 1). Failure pathways are analyzed in terms of sodium‐inventory loss and interfacial instability, followed by a reorganization of material strategies according to their functional roles in mitigating these processes. Performance metrics and scaling constraints are then examined to define practical boundary conditions, indicating the gap between laboratory evaluation and industrial requirements. Finally, advanced diagnostic and data‐driven approaches are discussed as tools for resolving interfacial complexity and enabling predictive design. Taken together, this Review establishes a system‐level framework for understanding and controlling sodium‐inventory‐limited batteries, providing a pathway toward their practical implementation.

**FIGURE 1:**
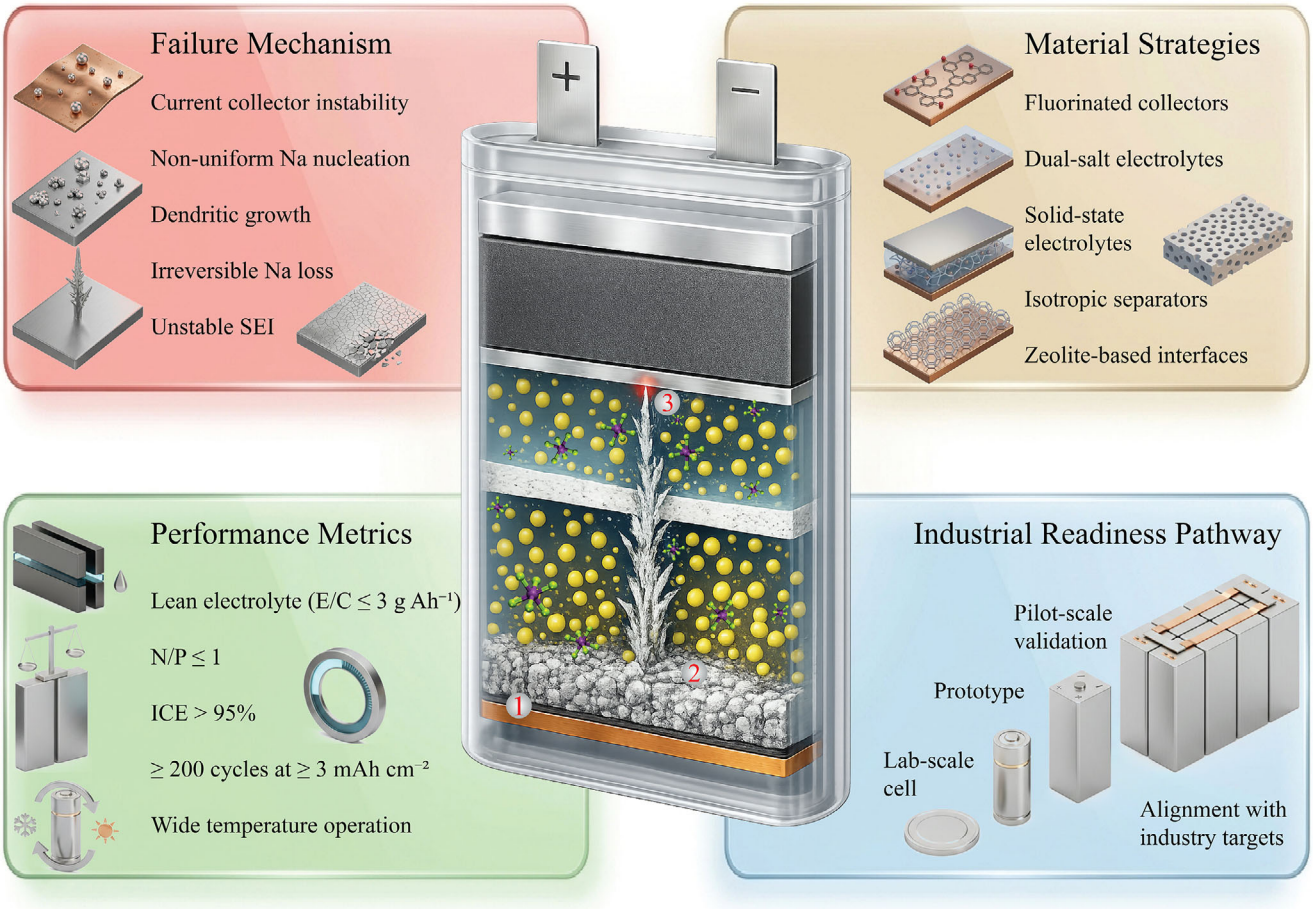
Conceptual framework for advancing anode‐free sodium metal batteries. A dissected pouch cell (center) anchors the framework, while the surrounding quadrants represent failure mechanisms, material strategies, performance metrics, and pathways toward industrial readiness.

## 2 Fundamentals of the Anode‐Free System

### 2.1 Working Principle

AFSMBs operate through a plating‐stripping mechanism in which sodium metal is generated in situ on a bare current collector during the first charge and reversibly removed during discharge. Unlike conventional sodium‐metal batteries with preloaded Na foil, AFSMBs rely entirely on Na extracted from the cathode, while the collector serves only as the deposition substrate. This configuration eliminates excess anode mass and improves both gravimetric and volumetric energy density. In the absence of a sodium reservoir, any irreversible loss directly depletes the available inventory (Section 2.2) [20]. During charging, Na^+^ ions deintercalate from the cathode and migrate through the electrolyte to the current collector, where they are reduced to metallic Na according to:

**Cathode**: Na_x_MO_y_ → Na_x‐1_MO_y_ + Na^+^ +e^−^
**Anode**: Na^+^ + e^−^ →Na^0^ (plated on the collector)

During discharge, the plated sodium is oxidized back to Na^+^ and reinserted into the cathode lattice, completing the cycle. Although this reaction pathway preserves the overall stoichiometry of sodium‐ion batteries, it replaces bulk intercalation at the anode with interfacial metal deposition. As a result, AFSMBs can, in principle, maximize energy density, but this advantage critically depends on achieving near‐complete reversibility of Na plating and stripping.

Initial sodium deposition is governed by heterogeneous nucleation at the electrolyte‐collector interface, where the nucleation barrier, determined by surface energy, lattice matching, chemical affinity, and local Na^+^ concentration, controls the density and distribution of nuclei. Inert or oxidized surfaces exhibit high nucleation overpotentials, leading to sparse and non‐uniform deposition, whereas sodiophilic surfaces promote more homogeneous nucleation. Simultaneously, electrolyte reduction defines the composition of the initial SEI, coupling interphase formation with nucleation dynamics. Because nucleation preferentially recurs at pre‐existing sites, early‐stage heterogeneity propagates during subsequent cycling. In sodium‐limited systems, such non‐uniform deposition and associated side reactions directly accelerate sodium loss, making dense and spatially uniform nucleation a key requirement for stable anode‐free (AF) operation [21].

### 2.2 Defining the Sodium Inventory

Because AFSMBs contain no sodium reservoir, performance is set by an inventory balance rather than by electrode capacity alone. Terminology in the literature is inconsistent, so we define three categories according to electrochemical accessibility and use them throughout this Review. Reversible sodium is metallic Na^0^ that remains electronically connected to the current collector and can be stripped and re‐plated in subsequent cycles. Dead sodium is metallic Na^0^ that is physically present but electronically disconnected from the collector or fully encapsulated by an insulating SEI, and is therefore inaccessible under normal operation. Chemically bound sodium is sodium consumed into SEI/CEI phases, electrolyte decomposition products, or crossover species, and is present in ionic rather than metallic form. Dead sodium and chemically bound sodium together constitute the irreversible inventory loss, and per cycle:

(1) $$Q_{\mathrm{plated}}=Q_{\mathrm{stripped}}+Q_{\mathrm{dead}}+Q_{\mathrm{chem}}$$

The distinction is not semantic. Chemically bound sodium scales with the fresh surface area generated each cycle and cannot be recovered once formed. Dead sodium is governed by deposit morphology, connectivity and mechanical stress, and is in principle partially recoverable if electronic contact is re‐established, as demonstrated for isolated lithium. The two channels therefore respond to different design levers and scale differently with areal capacity, current density and stack pressure, yet Coulombic efficiency reports only their sum. Equation (1) is experimentally verifiable. *Q*_stripped_ is the galvanostatic stripping capacity; (*Q*_stripped_ + *Q*_dead_) is the total residual Na^0^, accessible by ^23^Na NMR through the Knight‐shifted metallic resonance or by titration gas chromatography; Q_chem_ then follows by difference and can be cross‐checked by ICP‐OES and depth‐resolved XPS. Reporting the three terms separately, rather than Coulombic efficiency alone, would allow the dominant loss channel to be identified in a given system (Section 6). Cell configuration must be reported alongside these quantities. We use anode‐free (AF) for cells assembled on a bare collector with no sodium reservoir (N/P = 0), and anode‐less where a limited reservoir is present (0 –< N/P ≲ 2), whether pre‐deposited, pre‐sodiated, or supplied as a thin foil. Coulombic efficiencies obtained in Na||Cu half‐cells transfer to neither, since the reservoir buffers the very inventory loss that limits reservoir‐free operation. Configurations are indicated where performance is quoted and in Table 2.

### 2.3 Sodium Deposition Behavior and Electrochemical Metrics

Compared with lithium, sodium exhibits distinct physicochemical characteristics that fundamentally alter deposition behavior in AF systems (Figure 2a–c). Owing to its larger ionic radius and weaker Lewis acidity, Na^+^ has a lower desolvation barrier, enabling faster interfacial charge transfer but reducing control over nucleation and growth. Sodium typically exhibits higher nucleation overpotential and isotropic nucleation, resulting in three‐dimensional, porous morphologies rather than compact deposition (Figure 2b). These structures increase surface area and amplify current‐density heterogeneity, leading to higher polarization and instability. In parallel, sodium's higher chemical reactivity drives continuous electrolyte reduction, forming softer, organic‐rich SEIs that are mechanically fragile and dynamically reformed (Figure 2c). The result is a trade‐off of fast kinetics against poor morphological and interfacial stability, which accelerates inventory loss where no reservoir exists [22, 23].

**FIGURE 2:**
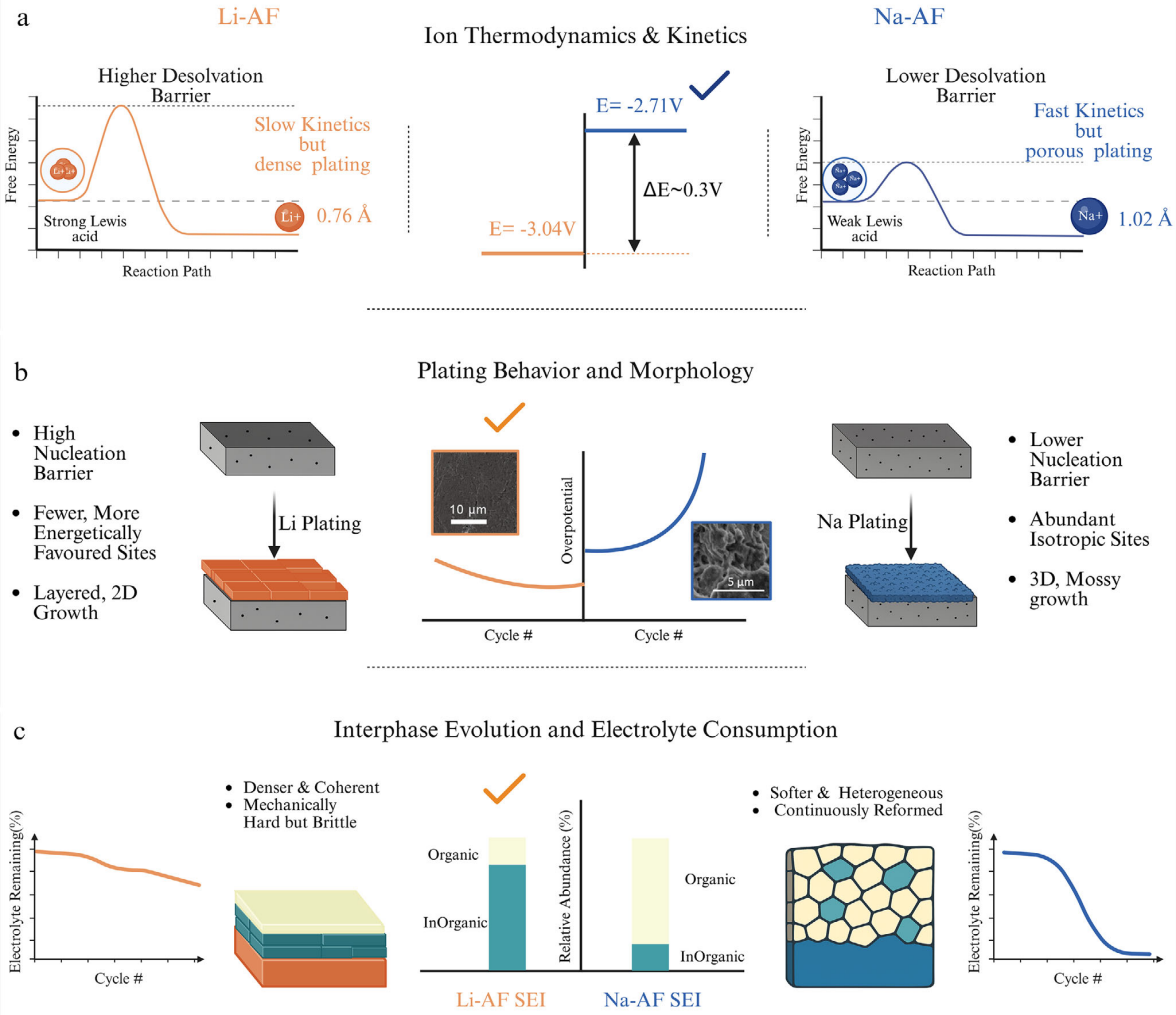
Key physicochemical distinctions between Li‐AF and Na‐AF systems. (a) Ion‐solvation and desolvation energy diagrams showing the weaker Lewis acidity and lower desolvation barrier of Na^+^. (b) Plating morphology illustrating higher nucleation overpotential and the tendency of Na to form 3D, mossy deposits. (c) Evolution of the interphase and electrolyte consumption, highlighting the formation of softer, heterogeneous, organic‐rich SEI layers that accelerate electrolyte loss. Both systems share dendrite and interphase challenges, but the larger size and higher reactivity of Na amplify these effects. Representative SEM image of Na (right) was adapted with permission from [26]. Copyright 2015, American Chemical Society. The Li (left) SEM image was adapted with permission from [32]. Copyright 2023, AIP Publishing.

Performance evaluation in AFSMBs extends beyond conventional metrics to parameters directly sensitive to sodium inventory, including initial Coulombic efficiency (ICE), average Coulombic efficiency (CE), areal capacity, and energy density. ICE quantifies irreversible sodium loss during the first cycle and sets the inventory available to all subsequent cycles; the commonly cited ≥90% threshold [24, 25, 26] is quantified below. Despite progress, most systems remain within 80–90% due to interphase formation, parasitic reactions, and collector instability. Engineered interfaces can improve ICE toward ≈95% in AF systems [19, 27, 28, 29], whereas solid‐state systems typically exhibit lower values (<85%) due to nucleation barriers and interfacial mismatch [30, 31]. However, inconsistent reporting and a lack of standardized testing protocols hinder meaningful comparison.

Beyond the initial cycle, average CE governs long‐term sodium retention. Sustained CE ≥ 99.9% over extended cycling is required to maintain ≈80% capacity retention at the cell level, showing the extreme sensitivity of AFSMBs to cumulative sodium loss. Representative systems have approached this threshold under controlled conditions [15, 33], yet fewer than ≈13% of reported studies achieve CE > 99.7% under practically relevant, lean‐electrolyte conditions, well below the requirement derived below (Table 1). Areal capacity further dictates scalability, with ≥3 mAh cm^−2^ widely recognized as the industrial benchmark [34, 35]. However, more than 80% of reported systems operate below 2 mAh cm^−2^, thereby underestimating the severity of interface instability and dendrite formation at practical loadings [36]. At the device level, current AFSMB pouch cells typically deliver 200–210 Wh kg^−1^, which remains 20–30% lower than Li‐ion counterparts (250–300 Wh kg^−1^) [37, 38]. This gap arises primarily from limited cathode capacities (<160 mAh g^−1^) and excessive electrolyte consumption (>5 g Ah^−1^), both of which reduce practical energy density [39]. Even advanced systems achieving high CE at moderate areal capacities often rely on elevated electrolyte volumes, underscoring the trade‐off between interfacial stability and energy density [40]. Collectively, these metrics establish that performance in AFSMBs cannot be assessed in isolation. A unified evaluation framework, integrating ICE, CE, areal capacity, and electrolyte consumption under standardized conditions (temperature, pressure, and N/P ratio), is essential for benchmarking true progress and guiding the transition toward industrially viable systems. More fundamentally, these performance limitations originate from the intrinsic deposition behavior, dynamic interphase evolution, and extreme sensitivity of sodium inventory in AF configurations, which collectively give rise to coupled degradation pathways and irreversible sodium loss.

**TABLE 1:** Minimum average Coulombic efficiency required for anode‐free operation. Values derived from R = ICE × CE^(n−1)^ for 80% capacity retention, where R is retention, ICE the initial Coulombic efficiency, and n the cycle number. At N/P = 0 no reservoir compensates inefficiency, so ICE and average CE cannot be optimized independently.

Target cycles (80% retention)	ICE = 100%	ICE = 95%	ICE = 90%
200	99.888%	99.914%	99.941%
500	99.955%	99.966%	99.976%
1000	99.978%	99.983%	99.988%

The Coulombic efficiency required for practical operation can be derived rather than assumed. At N/P = 0, every inefficiency removes inventory permanently, so capacity retention after n cycles is R = ICE × CE^(n−1)^, and the minimum average CE consistent with a retention target is CE_min_ = (R/ICE)^(1/(n−1))^. For 80% retention (Table 1), 200 cycles require ≥99.89% at ICE = 100% and ≥99.94% at ICE = 90%; 500 cycles require ≥99.96% and ≥99.98%; 1000 cycles require ≥99.98% and ≥99.99%. A first‐cycle loss of 10% must therefore be amortized by an additional ∼0.01%–0.05% on every subsequent cycle, which explains why ICE and CE cannot be optimized independently in AF cells. Measured against this criterion, the best reported values remain far from practical. A cell averaging 99.7% CE reaches 80% retention after ∼74 cycles, and 99.9% after ∼223 cycles, both below the 500‐cycle threshold generally required for commercial deployment. These figures are upper bounds, as the derivation assumes constant CE, no cathode‐side sodium loss, and no reactivation of dead sodium; in multilayer pouch cells, edge plating and electrolyte starvation lower the effective value further (Section 5). Sustained 500‐cycle AF operation therefore requires average CE above ∼99.96% under lean‐electrolyte conditions, nearly sevenfold lower loss per cycle than the current state of the art.

## 3 Failure Cascade in Sodium‐Inventory‐Limited Systems

Failure in AFSMBs originates at the interface and evolves through a coupled cascade of processes that progressively deplete the limited sodium inventory. In the absence of excess metallic Na, even minor inefficiencies during nucleation, growth, and stripping accumulate irreversibly, leading to rapid capacity decay and early cell failure. As illustrated in Figure 3, these degradation pathways are not isolated but form an interconnected cascade, spanning current‐collector instability, non‐uniform nucleation, heterogeneous growth, dead sodium formation, and progressive interphase degradation. This section follows this progression from the initial nucleation event to the formation of dead sodium and the breakdown of the SEI, highlighting how each stage amplifies subsequent failure and accelerates irreversible sodium loss.

**FIGURE 3:**
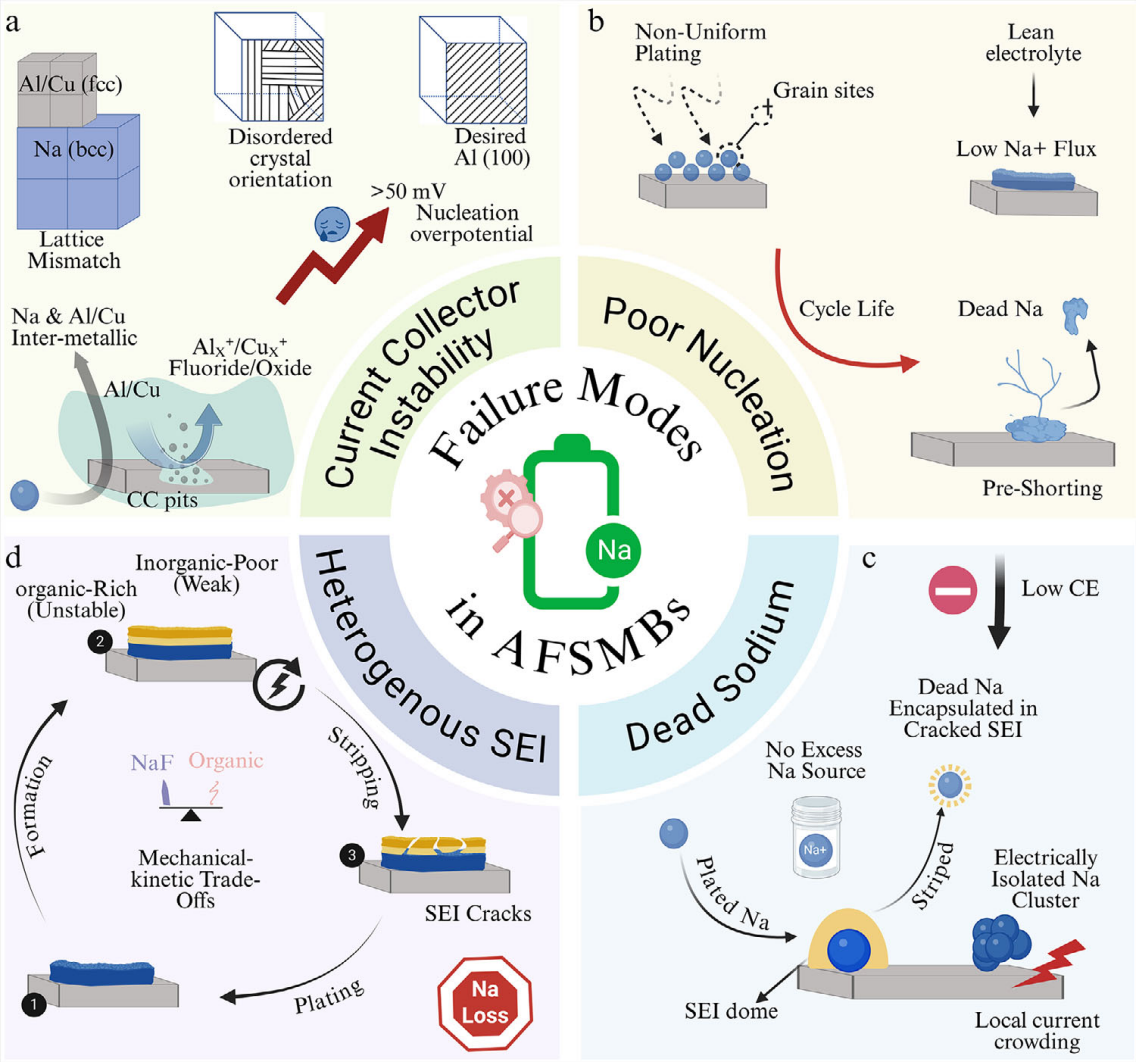
Interlinked failure mechanisms in anode‐free sodium metal batteries. (a) Current collector instability from lattice mismatch, surface oxides, and corrosion pits increases nucleation overpotential. (b) Poor nucleation under lean electrolyte leads to non‐uniform plating and early dead sodium formation. (c) Electrically isolated Na clusters (“dead sodium”) encapsulated in cracked SEI lower Coulombic efficiency and accelerate shorting. (d) Heterogeneous SEI evolution and mechanical‐kinetic trade‐offs cause repeated fracture, continuous SEI thickening, and progressive Na loss.

The current collector represents the initial site where sodium deposition is established and therefore plays a decisive role in determining the subsequent deposition behavior. In AFSMBs, the absence of a pre‐existing sodium layer makes nucleation highly sensitive to the surface chemistry and crystallographic structure of the collector (Figure 3a). Copper, although chemically stable, exhibits intrinsically low sodiophilicity, resulting in weak Na‐substrate interaction and a high nucleation overpotential. This promotes sparse and localized nucleation, leading to uneven initial sodium distribution and the formation of isolated deposits during early cycling [41]. Aluminum provides comparatively improved wettability and reduced nucleation barriers; however, lattice mismatch, surface oxides, and grain‐boundary heterogeneity still induce non‐uniform deposition and mechanical degradation upon repeated plating and stripping [15]. Surface defects, corrosion pits, and interfacial reactions further exacerbate these instabilities by introducing localized variations in current density [42].

Following current‐collector instability, the next stage is sodium nucleation, which determines the spatial distribution of deposited Na and governs subsequent growth behavior. Due to the intrinsically weak interaction between Na and most collector surfaces, nucleation typically occurs with a high overpotential, often exceeding 50 mV, significantly higher than that of lithium systems (≈10 mV) [43, 44]. Such values are protocol‐dependent: they are extracted variously from the initial voltage dip during galvanostatic plating, from the nucleation loop in cyclic voltammetry, or from the dip‐to‐plateau difference, and shift with current density, electrolyte composition, substrate history, and plating capacity [45]. Reported overpotentials are therefore comparable only within a common protocol, and the absolute values quoted throughout this Review should be read accordingly. As illustrated in Figure 3b, nucleation preferentially occurs at defects, grain boundaries, and high‐energy surface regions, leading to localized Na deposition rather than uniform coverage. This non‐uniform initiation establishes an uneven current distribution across the interface, which becomes further amplified under practical conditions. In particular, lean electrolyte environments and high areal capacities limit Na^+^ transport, creating localized flux deficiencies and promoting current crowding at active nucleation sites [46]. Nucleation heterogeneity is therefore not an initial inefficiency but a deterministic step, since the distribution of nuclei defines the subsequent growth pathways.

Once nucleation is established, sodium deposition proceeds through a growth process that amplifies any initial heterogeneity. Regions with higher local current density experience accelerated Na accumulation, while underutilized areas remain inactive, leading to progressively uneven deposition morphology. As shown in Figure 3b, this results in the formation of mossy, porous, or dendritic Na structures. Under lean electrolyte conditions, limited ionic transport further intensifies this effect by restricting Na^+^ replenishment at the interface. The imbalance between ionic flux and electron supply drives anisotropic growth, where sodium preferentially extends into regions of lower resistance. This leads to filamentous structures and irregular deposits that are mechanically fragile and poorly connected to the current collector. Such heterogeneous growth not only reduces the effective utilization of plated sodium but also introduces structural instability. Porous and loosely packed Na deposits are prone to fragmentation during stripping, setting the stage for the formation of dead sodium and irreversible capacity loss.

Following heterogeneous growth, irreversible sodium loss becomes the dominant degradation pathway in AFSMBs (Figure 3c). In the absence of excess sodium, every Na^+^ extracted from the cathode must be efficiently cycled. Any loss of electrical connectivity or chemical consumption directly reduces the available sodium inventory, leading to rapid capacity decay. Two primary mechanisms govern this process. First, mechanical disconnection during stripping generates dead sodium, nucleated by uneven plating and local current crowding, which accumulates over repeated cycles [10, 47]. Second, parasitic chemical reactions further consume active sodium. Metallic Na readily reacts with trace impurities such as moisture, forming NaOH and H_2_, which introduce porosity and structural defects. Additionally, electrolyte decomposition, particularly the hydrolysis of NaPF_6_, generates reactive species such as PF_5_ and HF that attack the interphase and accelerate its degradation [48]. The resulting chemical instability continuously consumes both sodium and electrolyte, compounding irreversible losses [49, 50, 51]. Together, these processes transform initially active sodium into dead sodium, progressively disconnecting the plating interface and driving capacity fade. Importantly, the extent of dead sodium accumulation remains difficult to quantify directly. Direct quantification of dead sodium remains rare, and the loss channels in Equation (1) are seldom separated experimentally (Section 6.1), given the sparse quantitative assessment of dead sodium in the literature [52, 53, 54, 55]. However, establishing a direct correlation between dead sodium volume and electrochemical performance remains an open challenge. Bridging this gap is essential for developing predictive models of AFSMB degradation and for identifying the dominant pathways of sodium loss. While dead sodium accumulation is a direct manifestation of anode‐side inefficiency, irreversible sodium loss in AFSMBs is not governed by anode processes alone.

Beyond anode‐side instabilities, cathode‐driven degradation constitutes an additional and often underappreciated failure pathway in AFSMBs. At elevated operating voltages, especially above ≈4.0 V versus Na^+^/Na, layered and polyanionic cathodes accelerate electrolyte oxidation and destabilize the cathode‐electrolyte interface, leading to the growth of thick, resistive CEI layers that hinder Na^+^ transport and promote capacity decay. In ether‐based systems, this instability can be severe enough to trigger rapid overcharge failure, whereas interphase‐regulating electrolytes suppress such decomposition and maintain thinner CEIs with lower interfacial resistance. Experimental studies further show that high‐voltage cathode degradation is accompanied by structural disorder or phase transition at the cathode surface, indicating that the failure is not limited to electrolyte consumption alone but also involves deterioration of the active host lattice [22, 23]. Crucially, these cathode‐side reactions are not spatially confined to the cathode. XPS analysis in cooperative‐interface AFSMB systems directly detected Ni and Fe species on the cycled Na anode, and their suppression after NaBF_4_ addition was attributed to the formation of a more protective CEI, providing direct evidence of transition‐metal crossover from cathode to anode. This cross‐electrode coupling implies that oxidative cathode degradation can propagate into anode‐side interfacial contamination, aggravate sodium loss, and accelerate Coulombic‐efficiency decay. Related electrolyte‐engineering studies also show that thinner, inorganic‐rich CEIs correlate with reduced transition‐metal dissolution and improved high‐voltage stability, reinforcing the view that cathode failure in AFSMBs should be understood as a coupled CEI‐SEI problem rather than an isolated cathode issue [40, 55].

Finally, among the major interfacial failure modes, the SEI in AFSMBs continuously reforms on freshly deposited sodium and therefore governs long‐term durability (Figure 3d). Three interconnected factors underpin its instability. First, dynamic mechanical failure arises from large volumetric fluctuations during Na plating/stripping, where repeated expansion and contraction fracture the SEI, expose fresh Na, and continuously consume both sodium and electrolyte, leading to progressive interfacial resistance growth [44, 56]. Second, compositional instability limits effective passivation. In contrast to Li systems, Na‐SEIs are typically dominated by organic components (≈60%–80%, such as Na_2_CO_3_ and NaOR), which exhibit inferior thermal and electrochemical stability and accelerate degradation [55, 57, 58]. Third, a persistent mechanical‐kinetic trade‐off constrains SEI optimization: inorganic‐rich (NaF‐dominated) interphases provide chemical robustness but require strong fluorination, whereas organic‐rich layers offer flexibility at the expense of long‐term stability [59, 60]. These limitations drive continuous SEI thickening, rising interfacial resistance, and progressive sodium consumption [61], establishing the SEI as the primary bottleneck for long‐term AFSMB operation.

This trend, however, should not be read as a simple ranking in which inorganic components are uniformly superior. Reported SEI compositions are not exclusively organic or inorganic but coexisting, and their relative fractions vary with salt chemistry, salt concentration, additive content, current density, and temperature, so that compositions determined under one set of conditions are not transferable to another [19]. Two observations from the sodium literature indicate that the relationship between inorganic content and reversibility is less direct than commonly assumed. First, ether‐based electrolytes such as 1 M NaPF_6_ in diglyme have been reported to sustain Coulombic efficiencies above 99.9% without fluorinated additives or elevated salt concentrations [46], although at this concentration the solvation structure is described as solvent‐separated‐ion‐pair dominated [23], rather than the contact‐ion‐pair and aggregate regimes from which anion‐derived interphase design was originally rationalized. Second, bulk alkali‐metal fluorides are among the least ion‐conductive phases identified in the interphase [62], so a strictly NaF‐dominated layer would be expected to impede rather than facilitate Na^+^ transport; reported reconciliations invoke transport along grain boundaries and heterointerfaces rather than through the bulk fluoride phase [63]. Reported characterization of glyme‐derived interphases has also not consistently identified the phosphorus‐containing decomposition products expected from extensive anion reduction [64].

Taken together, these observations do not overturn the correlation between inorganic‐rich interphases and improved reversibility, which is reproduced across many systems, but they indicate that inorganic content alone is an incomplete descriptor and that its causal role remains to be established. A more defensible design target is compositional balance: sufficient inorganic content for chemical and mechanical stability, sufficient organic or amorphous content for compliance under the volume changes of plating and stripping, and a continuous Na^+^ transport pathway through the layer. Discriminating cause from correlation will require comparisons at matched areal capacity, current density, and electrolyte‐to‐capacity ratio across electrolytes spanning distinct solvation regimes, together with measurements of transport through the interphase itself rather than inference from composition.

## 4 Mechanism‐Driven Intervention Strategies

Stabilizing sodium deposition in AFSMBs requires coordinated control of all interfacial boundaries rather than isolated optimization of individual components. In sodium‐limited architectures, localized instabilities cannot be buffered by excess Na and therefore propagate across the cell, leading to cumulative degradation. As illustrated in Figure 4a,b, this behavior can be understood as a coupled failure cascade, spanning (i) current‐collector instability, (ii) high nucleation overpotential, (iii) heterogeneous sodium growth, (iv) dead sodium formation with interphase fracture, and (v) crosstalk‐induced sodium depletion. Building on these interfacial limitations, materials design has evolved toward multi‐scale control of sodium deposition. At the atomic scale, interfacial chemistry can be tuned to reduce nucleation barriers, for example, through fluorinated motifs that lower nucleation overpotential below 10 mV [65]. At the mesoscale, three‐dimensional architectures redistribute ion flux and mechanical stress, enabling more uniform plating [66]. At the molecular level, electrolyte design regulates solvation structure and interphase composition, such as weakly solvating systems that promote inorganic‐rich SEI formation [22]. Solid‐state architectures provide an additional route by physically constraining dendritic growth and minimizing interfacial reactivity [30].

**FIGURE 4:**
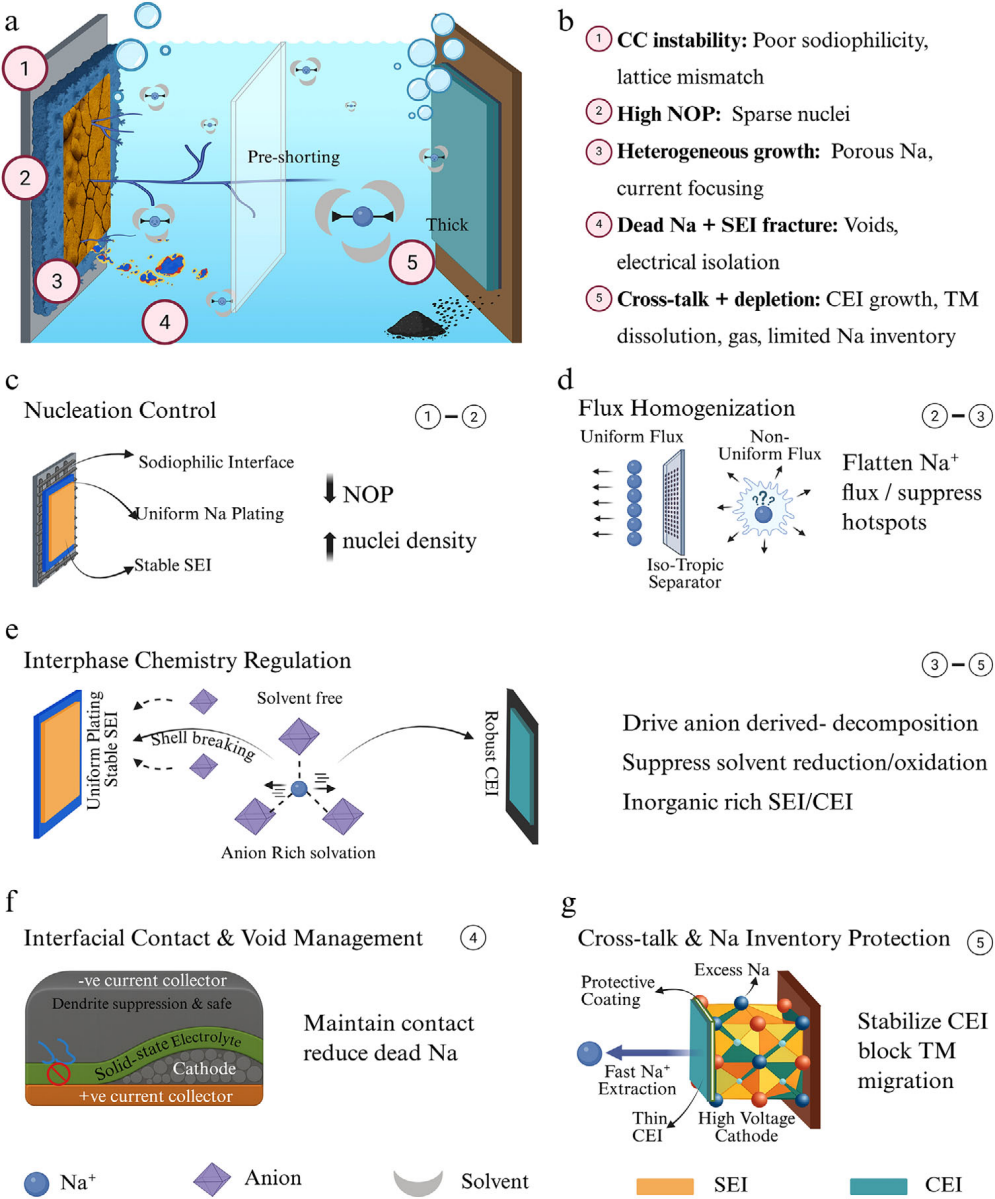
Failure cascade and functional intervention map for anode‐free sodium‐metal batteries. (a) Schematic of coupled degradation processes in AFSMBs, annotated as a failure cascade. (b) Failure‐cascade definitions linking each stage to its dominant physicochemical origin. (c‐g) Conceptual “functional intervention points” illustrating how representative material and interface strategies interrupt specific stages of the cascade: (c) nucleation control (1, 2), (d) ion‐flux homogenization (2, 3), (e) anion‐derived interphase programming to regulate SEI/CEI chemistry (3, 5), (f) interfacial contact and void management to suppress dead sodium accumulation (4), and (g) crosstalk mitigation and Na‐inventory protection via CEI stabilization and transition‐metal (TM) blocking (5).

Accordingly, the following sections analyze material strategies through the functional intervention map defined in Figure 4c–g, linking specific design levers to the stages of the failure cascade. Advances are organized into five domains: interphase chemistry, nucleation and growth, sodium inventory and crosstalk, solid‐state interfaces, and ion transport regulation, with emphasis on mechanistic cause‐and‐effect relationships and their practical implications.

### 4.1 Interphase Chemistry

To suppress nucleation heterogeneity, dead sodium formation, and SEI/CEI instability, electrolyte design in AFSMBs has evolved from empirical formulation to mechanism‐guided interphase control. In AFSMBs, the electrolyte governs more than Na^+^ transport as it defines the solvation environment, directs interphase reaction pathways, and determines how much sodium and solvent are irreversibly consumed during cycling. Unlike sodium‐metal cells containing excess Na, AF configurations cannot compensate for Na^+^ lost during early SEI formation or isolated deposition. Electrolyte‐driven inefficiencies therefore become directly cycle‐limiting at practical loadings, requiring simultaneous control of interfacial thermodynamics and transport kinetics to preserve sodium inventory and reversibility [67, 68]. Recent advances move beyond empirical salt‐solvent selection toward mechanism‐guided regulation [69]. Figure 5 summarizes these control modes and their relationship to the AF failure cascade. Rather than classifying electrolytes by composition, it is more informative to differentiate them by their dominant physicochemical lever, whether they bias decomposition toward anion‐derived interphases, regulate solvation structure through contact ion pair (CIP) and higher‐order aggregate (AGG) control, immobilize reactive anions to redistribute ionic flux, program interphase chemistry via selective bonding and orbital alignment, impose geometric confinement on solvation clusters, or stabilize salt dissociation through entropic effects [65].

**FIGURE 5:**
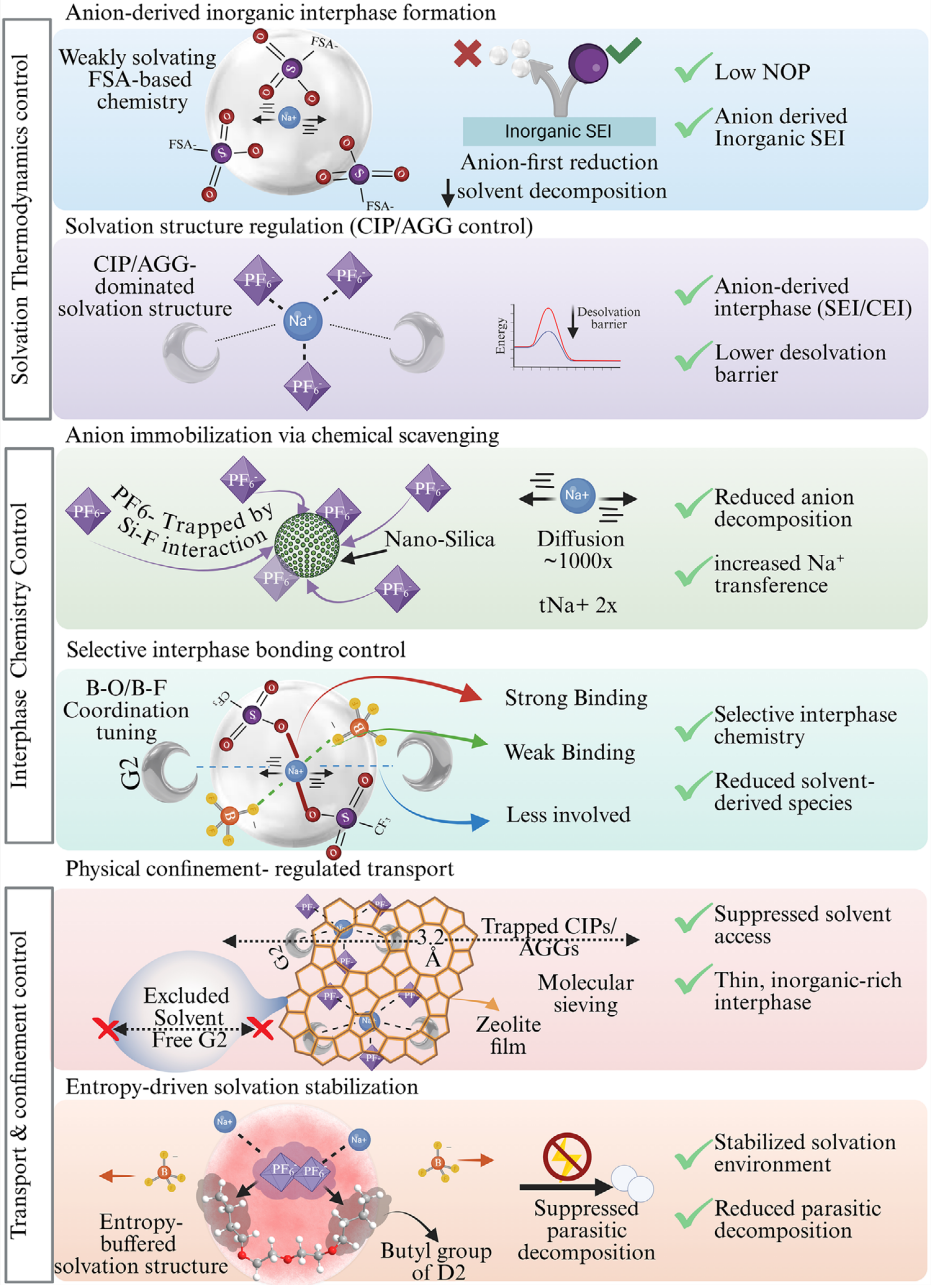
Mechanistic framework of electrolyte engineering in anode‐free sodium metal batteries. Six control mechanisms across three engineering domains: anion‐first interphase formation, solvation thermodynamic regulation (CIP/AGG control), anion immobilization via chemical scavenging, bonding‐environment modulation, transport regulation through physical confinement, and entropy‐driven solvation stabilization. Each regulates interphase composition, Na^+^ desolvation, ion flux distribution, and deposition morphology through a distinct physicochemical lever [21, 22, 23, 40, 65, 70, 71].

At the most fundamental level, electrolyte design can bias interphase chemistry toward anion‐derived inorganic products. In solvent‐dominated carbonate or ether systems, solvent molecules preferentially reduce during early Na plating, forming organic‐rich SEIs that are mechanically unstable and continuously consume active sodium. Dual‐salt weak‐solvation electrolytes demonstrate that rational salt selection can invert this hierarchy [21, 55, 70]. Substituting PF_6_^−^ with BF_4_^−^ or OTF^−^ modifies Na^+^‐solvent coordination and promotes preferential anion participation in interphase formation, as evidenced by radial distribution functions and vibrational spectroscopy. Cooperative‐interface strategies further exploit frontier orbital alignment to regulate sequential redox processes, in which one salt decomposes preferentially at the anode while another contributes to cathode‐side CEI formation [10]. Depth‐resolved XPS, ToF‐SIMS mapping, and cryogenic microscopy show thinner, B‐O/B‐O‐F‐enriched interphases with reduced structural heterogeneity. Although both approaches generate inorganic‐rich SEI/CEI layers, weak‐solvation systems primarily alter reduction pathways through coordination effects, whereas cooperative‐interface designs program decomposition sequences through electronic hierarchy. In each case, directing interphase chemistry toward anion‐derived inorganic species suppresses solvent‐driven side reactions and improves sodium reversibility under AF conditions.

Beyond the decomposition hierarchy, AFSMB performance is strongly governed by Na^+^ solvation structure. Regulating the balance between solvent‐separated ion pairs, CIPs, and AGGs reshapes both bulk transport and interfacial desolvation behavior. Electrolytes that shift the association‐dissociation equilibrium toward CIP‐rich configurations increase anion participation within the primary solvation shell, reduce Na^+^ coordination with ether oxygen species, and alter interfacial reaction kinetics. Spectroscopic analyses and molecular simulations indicate that such CIP/AGG‐enriched structures persist near electrode surfaces during cycling, facilitating inorganic‐rich SEI and CEI formation while reducing reliance on solvent reduction [22]. In several systems, this solvation restructuring correlates with lower calculated desolvation energies and improved low‐temperature kinetics. Unlike redox‐sequence control, which programs which species decompose first, solvation‐structure regulation modifies how Na^+^ sheds its coordination shell at the interface, thereby reducing nucleation overpotential and promoting more uniform deposition under cryogenic or high‐rate conditions.

A mechanistically distinct strategy involves immobilizing reactive anions through chemical scavenging. In nano‐silica‐modified electrolytes, surface Si‐OH groups interact with PF_6_^−^ via Si‐F bonding, reducing anion mobility and lowering the anion transference number [71]. Preferential PF_6_^−^ adsorption promotes more complete Na^+^ dissociation and increases the Na^+^ transference number, leading to higher effective Na^+^ conductivity despite a modest decrease in bulk ionic conductivity. Chronoamperometric analysis interpreted through the Scharifker–Hills model reveals a transition from three‐dimensional instantaneous nucleation in conventional electrolytes to progressive nucleation in the suspension system. The reported increase in Na^+^ diffusion coefficient, approaching three orders of magnitude, delays Sand's time and mitigates concentration polarization during deposition. In contrast to solvation‐structure regulation, this mechanism operates by constraining anion transport rather than altering Na^+^ coordination chemistry, redistributing ionic flux to promote more uniform sodium growth.

Selective interphase bonding control represents another distinct lever. In dual‐salt weak‐solvation systems, modifying anion chemistry alters frontier orbital energies and the relative reduction/oxidation sequence of electrolyte components. LUMO/HOMO analysis indicates that certain salts preferentially decompose at the anode while remaining stable at the cathode, or vice versa, enabling controlled formation of compositionally distinct SEI and CEI layers [10]. Spectroscopic and electronic‐structure analyses show that tailored bonding environments weaken specific Na^+^ solvent interactions and adjust electrostatic potential distributions, influencing how interphase species nucleate and organize. Unlike CIP/AGG regulation, which modifies association states within the solvation shell, selective bonding control programs interphase chemistry through electronic design, governing decomposition sequence and chemical uniformity at both electrodes [21, 55, 70].

Physical confinement introduces spatial regulation into electrolyte design. In zeolite‐modified systems, a 3A molecular sieve film with ≈3.2 Å pores imposes geometric constraints on solvated species, selectively trapping aggregated solvation structures while permitting Na^+^ transport [23]. This size‐exclusion effect promotes aggregation‐dominated electrolyte configurations within confined domains and suppresses continuous oxidative decomposition, extending oxidative stability to higher voltages without compromising Na plating/stripping reversibility. Confinement‐regulated transport stabilizes interphases by spatially restricting ion transport, decoupling solvent accessibility from ion transport under high‐voltage operation. A conceptually distinct pathway emerges from entropy‐driven solvation stabilization. In sole‐solvent systems based on diethylene glycol dibutyl ether (D2), weak Na^+^ solvating ability combined with enhanced anion‐solvent interactions produces elevated solvation entropy, as evidenced by temperature‐dependent electrochemical measurements. Molecular dynamics simulations reveal an anion‐rich primary solvation shell with increased PF_6_^−^ coordination relative to conventional ether systems [40]. This entropy‐assisted salt dissociation offsets reduced cation‐solvent coordination, enabling high salt solubility despite a low dielectric constant. The resulting solvation structure exhibits lower calculated desolvation energies and maintains stability across a wide temperature range (−20 to 60 °C). Entropy‐driven stabilization promotes species diversity and configurational disorder rather than adjusting association fractions, thermodynamically favoring stable anion‐derived interphases and wide‐temperature ionic transport.

These electrolyte strategies demonstrate that interphase stabilization in AFSMBs arises from multiple, partially orthogonal controls rather than a single dominant variable. Decomposition hierarchy and selective bonding reprogram interphase chemistry at the electronic level, solvation‐structure engineering and entropy stabilization reshape Na^+^ coordination thermodynamics, and chemical scavenging or confinement regulate mass transport through spatial and flux redistribution. Integrating compatible control modes, rather than optimizing any single parameter in isolation, will be essential to minimize sodium loss across the AF failure cascade. The mechanistic distinctions outlined above are substantiated by converging computational, spectroscopic, and operando characterizations across representative AFSMB systems (Figure 6). Frontier orbital calculations, molecular dynamics simulations, vibrational spectroscopy, and depth‐resolved interphase analyses collectively clarify how electrolyte modifications reshape Na^+^ coordination environments, decomposition pathways, and SEI/CEI architectures under realistic conditions. Such multi‐modal validation reinforces that electrolyte performance in AFSMBs reflects controlled physicochemical processes rather than empirical formulation alone. In this context, electrolyte engineering establishes the chemical boundary conditions for sodium deposition, while subsequent materials strategies, such as current collector modification and interfacial structuring, operate within and respond to this electrolyte‐defined landscape [72].

**FIGURE 6:**
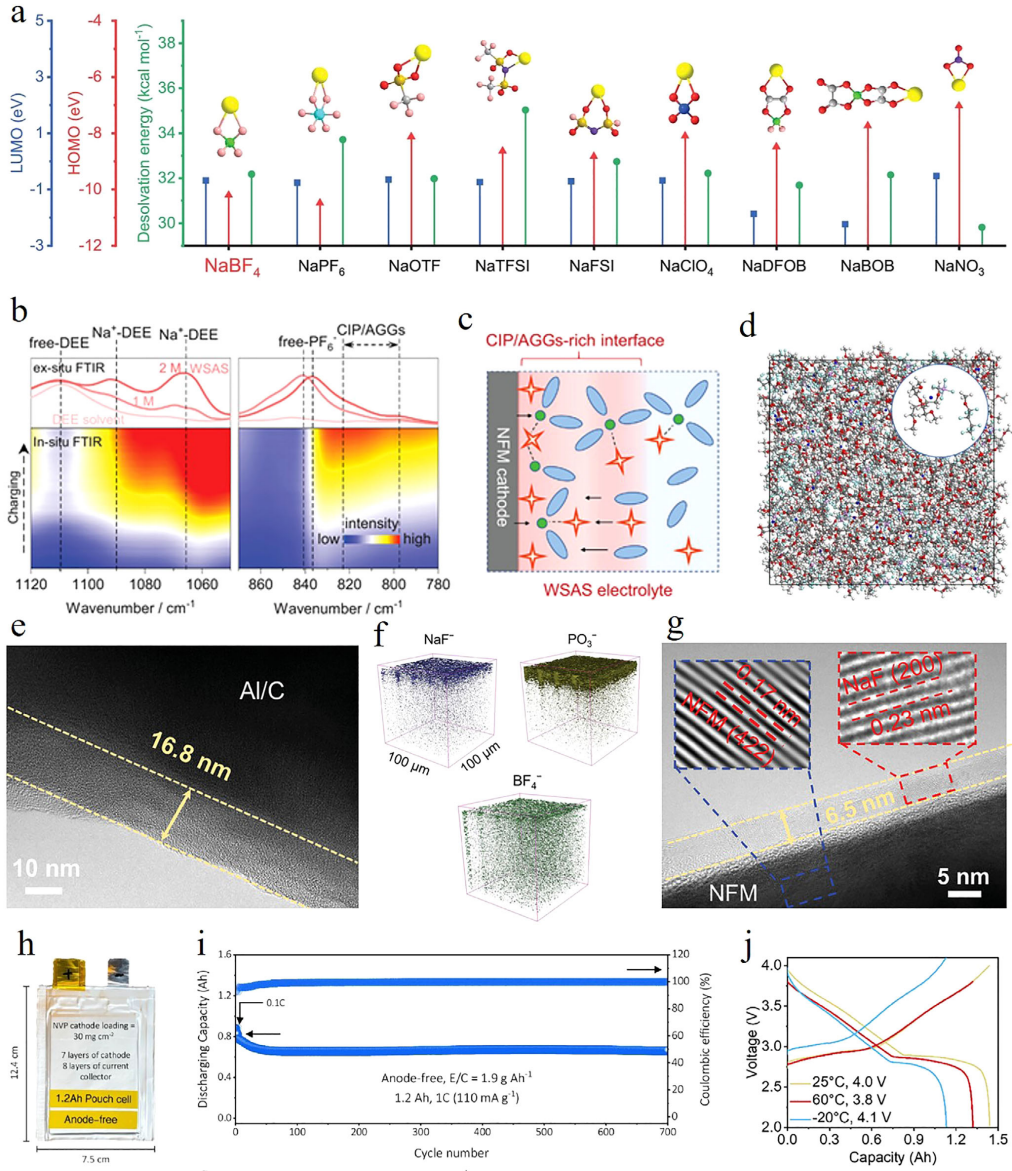
Electrolyte engineering strategies for stable interphases in AFSMBs. (a) Computational screening of salt and additive properties guiding electrolyte formulation (Adapted with permission from [70]. Copyright 2025, Oxford Academic). (b) Operando FTIR of contact ion pairs (CIPs) and aggregates (AGGs) formation during charging. (c) Anion‐rich solvation structures at electrode interfaces (Adapted with permission from [22]. Copyright 2024, Wiley). (d) Molecular dynamics of fluorinated solvent systems (Adapted with permission from [48]. Copyright 2025, Springer Nature). (e‐g) Interphase characterization: cryo‐TEM of SEI nanostructure, ToF‐SIMS depth profile indicating inorganic‐rich composition, and TEM with IFFT of the crystalline CEI (Adapted with permission from [40]. Copyright 2025, Wiley); see Table 5 for the artifact limitations of each. (h‐j) Electrochemical validation:1.2 Ah pouch cell and cycling at 1C (Adapted with permission from [48]. Copyright 2025, Springer Nature), and operation from −20 to 60°C (Adapted with permission from [40]. Copyright 2025, Wiley).

### 4.2 Nucleation and Growth

In AFSMBs, sodium nucleation and subsequent growth define the initial conditions for deposition morphology and ultimately determine the reversibility of stripping. Owing to sodium's weak affinity for conventional current collectors such as copper or aluminum, nucleation is typically heterogeneous, leading to uneven growth, unstable interphase formation, and progressive sodium loss. These early‐stage instabilities are particularly detrimental in AF architectures, where no excess sodium reservoir exists to compensate for inactive Na accumulation. Consequently, controlling nucleation thermodynamics and growth kinetics is central to stabilizing sodium deposition. As summarized in Figure 7, current collector and interface engineering strategies can be broadly categorized into six mechanistic classes: fluorinated interlayers, three‐dimensional architectures, alloy‐based interfaces, plasma‐modified surfaces, chemically functional coatings, and crystallographic or compositional engineering approaches. Each operates through a distinct physicochemical lever to regulate nucleation behavior, electric‐field distribution, and interfacial stress evolution under lean‐electrolyte and high‐loading conditions.

**FIGURE 7:**
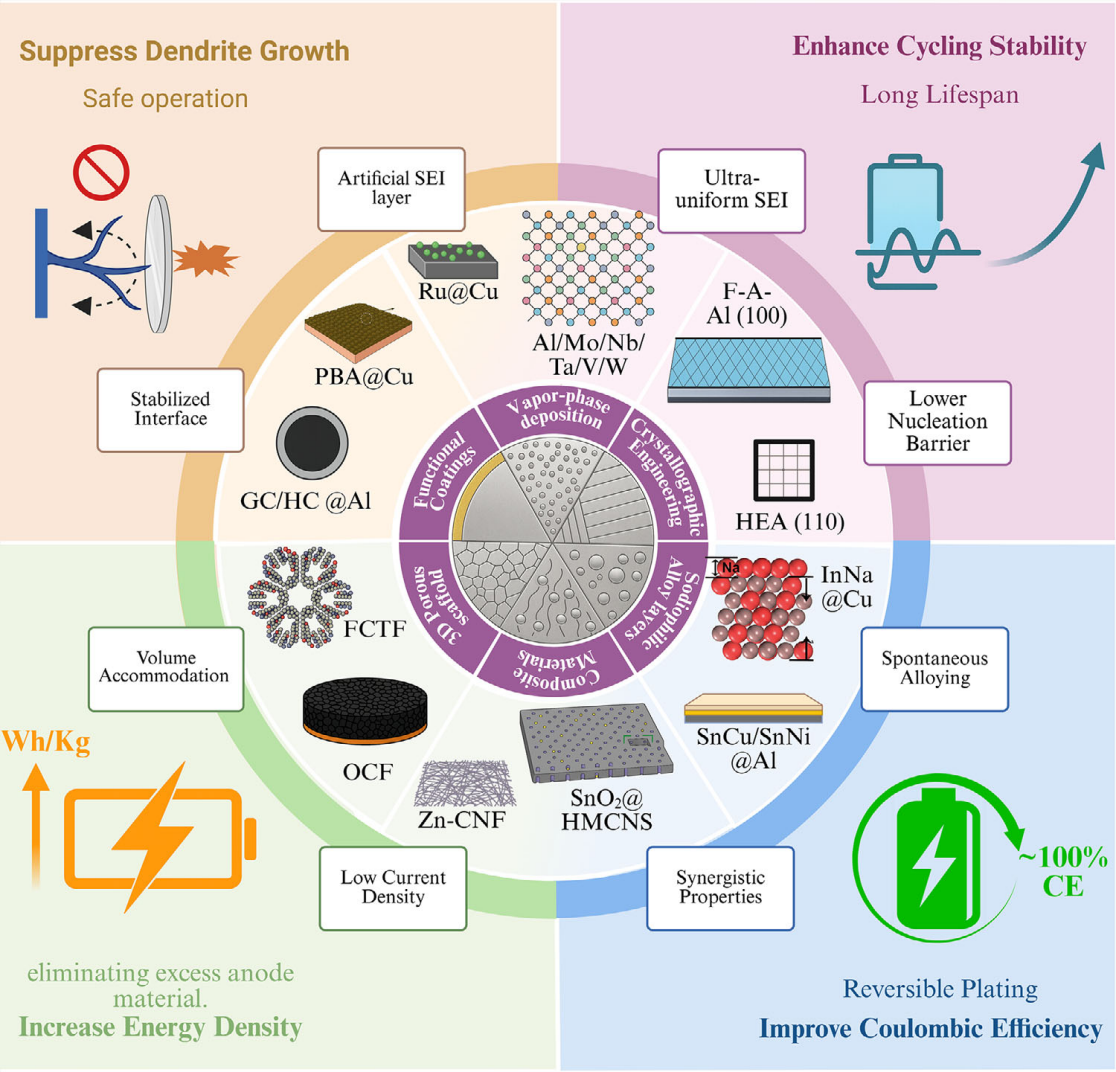
Multi‐level current collector engineering for anode‐free sodium‐metal batteries. A segmented current collector at center, surrounded by the six material design classes defined in the text, each shown with a representative implementation from the literature.

Fluorinated interlayers are among the most effective chemical approaches for stabilizing sodium deposition in AFSMBs. Their central mechanism is the in situ formation of NaF‐rich interphases, which offer high mechanical modulus and chemical stability. By introducing fluorine at the collector interface, nucleation thermodynamics are reprogrammed toward inorganic‐dominated SEI formation, suppressing solvent‐driven side reactions and reducing irreversible sodium consumption. Our recent work on a fluorine‐integrated monolithic conductor (FCNF) demonstrates this route [73]. Controlled solvothermal fluorination of a carbon nanotube framework introduces semi‐ionic C‐F bonds and expands the interlayer spacing to ≈0.42 nm (Figure 8a), serving three functions: polarized C^+^‐F^−^ bonds confer sodiophilicity, the expanded channels facilitate Na^+^ diffusion, and the lattice acts as an integrated fluorine source for a thin NaF‐rich SEI. Cycling in a fluorine‐free electrolyte (NaClO_4_) with ToF‐SIMS depth profiling indicated that the fluorine in this SEI originates from the scaffold rather than the salt anion (Figure 8b), showing that NaF formation need not proceed through anion decomposition. The resulting interphase supports reversible plating and stripping (>99.9% CE over 800 cycles at 3 mAh cm^−2^) and 94.2% retention over 150 cycles in an AF pouch cell. That the SEI remains thin is relevant here, since a thick NaF‐dominated layer would impose a transport penalty given the low ionic conductivity of bulk NaF (Section 3).

**FIGURE 8:**
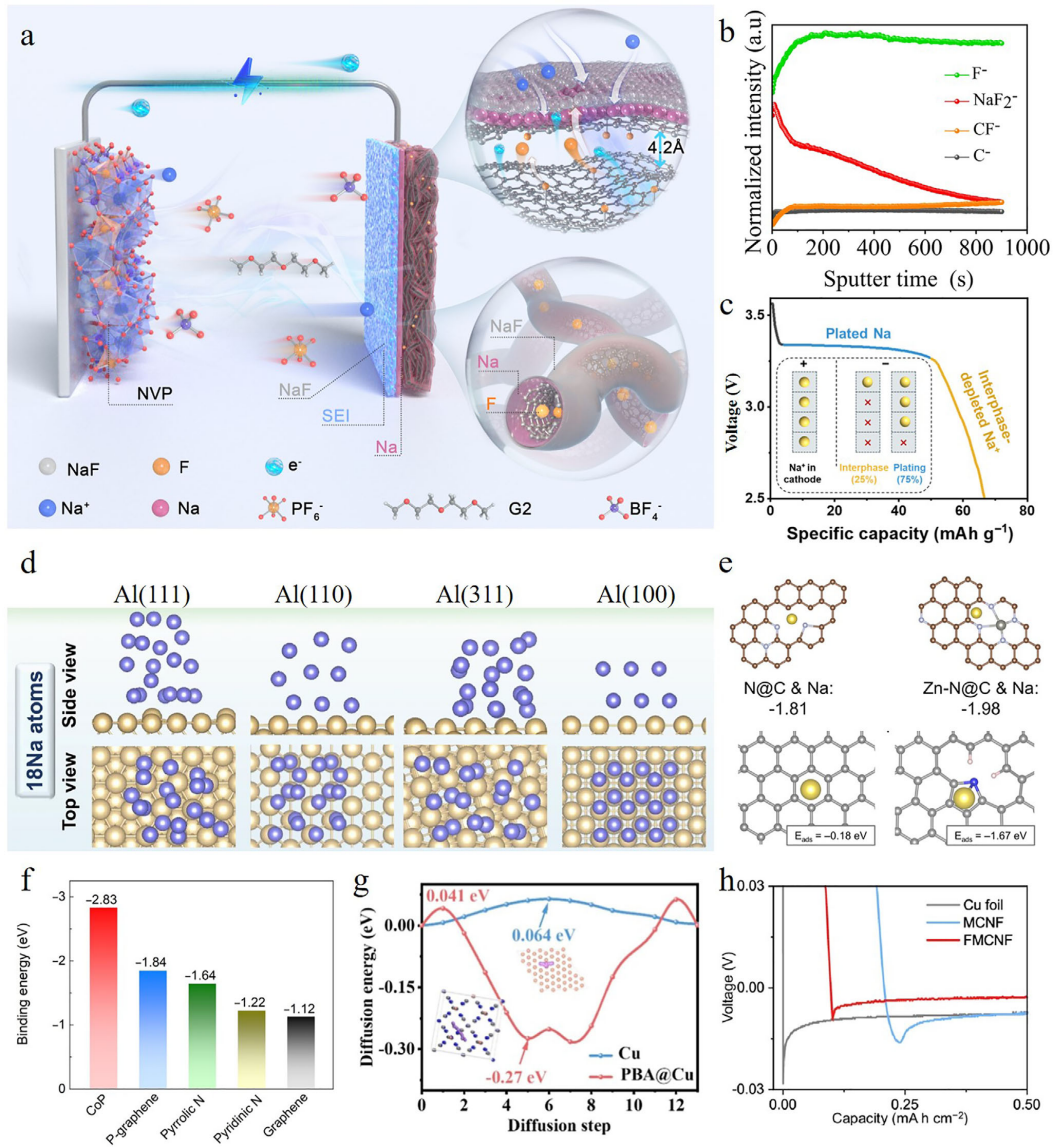
Atomic‐level design of sodiophilic current collectors. (a) Schematic of the FCNF architecture: semi‐ionic C‐F bonds provide sodiophilic nucleation sites and expanded ion channels, while lattice fluorine acts as an in situ donor for a NaF‑rich SEI. (b) ToF‑SIMS depth profiles after cycling in fluorine‑free electrolyte (NaClO_4_). The NaF_2_^−^ signal is confined to the outer interphase, whereas CF^−^ extends throughout the scaffold, indicating that lattice fluorine is the origin of the NaF‐dominated interface (Adapted with permission from [73]. Copyright 2026, Wiley). (c) Quantitative Na distribution showing high plating efficiency due to a fluorinated interface (Adapted with permission from [44]. Copyright 2023, AAAS). (d) Simulated 18 Na atoms adsorption behavior on different Al crystal facets (Adapted with permission from [15]. Copyright 2025, Springer Nature). (e, f) Computational and experimental binding energies of Na on various carbon‐based substrates. (Panel e adapted with permission from [21, 27]. Copyright 2025, KeAi (top) and copyright 2024,Cell Press (bottom). Panel f adapted With permission from [80]. Copyright 2025, AAAS) (g) Na^+^ diffusion energy barriers and corresponding pathways on Cu and a Prussian blue analogue‐coated Cu (PBA@Cu) (Adapted with permission from [41]. Copyright 2025, American Chemical Society). (h) Measured nucleation overpotential for different current collector modifications(Adapted with permission from [66]. Copyright 2025, Springer Nature). Values are protocol‐dependent and comparable only within the measurement conditions of the original studies.

The broader applicability of this fluorine‐chemistry lever is evident across other systems. For example, in fluorinated carbon frameworks such as FCTF, well‐defined fluorine‐rich edge sites provide strong sodiophilicity while maintaining moderate interfacial reactivity (Figure 8c), enabling highly reversible Na plating with an average CE of 99.6% over 400 cycles and supporting anode‐less pouch cells reaching 325 Wh kg^−1^ with 94.7% capacity retention after 300 cycles [44]. Similarly, fluorine‐doped mesoporous carbon nanofibers (FMCNFs) integrate micropore‐mesopore confinement with electronic modulation through Zn‐N_x_ coordination, sustaining over 5000 half‐cell cycles at 5 mA cm^−2^ with an average CE of 99.93% [66]. Fluorination has also been directly applied to metallic foils. Beyond carbon hosts, fluorination of metallic collectors further demonstrates the universality of this mechanism. Fluorine‐enriched (100)‐oriented aluminum surfaces provide lattice‐matched nucleation sites and promote NaF‐dominant SEI formation (Figure 8d), delivering ≈98% CE in AF configurations without activation protocols [65]. Across these systems, fluorination operates not merely as a surface coating but as an interfacial chemical regulator that lowers nucleation barriers, homogenizes sodium flux, and reinforces SEI robustness (Figure 8e–h). The consistency of improved sodium utilization and extended cycling across carbon and metallic platforms shows fluorine chemistry as a transferable design lever in current collector engineering.

Beyond interfacial chemistry, the topology of the collector shapes sodium deposition by redistributing local current density and buffering volumetric expansion. Three‐dimensional (3D) architectures mitigate current amplification at protrusions, lower effective nucleation barriers, and spatially homogenize Na^+^ flux (Figure 8d,e). Porous frameworks reduce localized field concentration, thereby suppressing dendritic growth and stress‐induced fracture during repeated cycling. Hierarchically porous carbon frameworks represent the dominant class of 3D hosts. Through tailored pore networks and heteroatom doping, these materials integrate structural confinement with electronic modulation. Nitrogen‐ or oxygen‐doped carbons and metal‐nitrogen coordinated sites provide distributed sodiophilic nucleation centers, while the porous backbone accommodates plating‐induced expansion (Figure 8g). Representative systems have demonstrated energy densities exceeding 330–370 Wh kg^−1^ and sustained cycling beyond hundreds to over a thousand cycles in optimized AF configurations [27, 74, 75, 76]. Metallic and alloy‐based 3D scaffolds offer complementary advantages, including intrinsic sodiophilicity and greater mechanical stiffness. Architectures such as bismuth nanosheets [77], copper phosphide arrays [78], and zinc‐modified aluminum meshes [79] promote uniform wetting and planar growth under practical loadings. Hybrid carbon‐metal systems combine electronic conductivity, catalytic functionality, and mechanical compliance, as demonstrated by cobalt phosphide nanoparticles in N, P‐doped carbon fibers, achieving >99.97% CE in Na plating/stripping at 10 mA cm^−2^ and 10 mAh cm^−2^ [80], tin dioxide‐decorated hollow carbon nanosheets retaining ≈80% capacity after 200 cycles (AF coin cell) [81], and iron‐carbon nanoparticles preserving 103 mAh g^−1^ over 100 cycles at high loading (AF coin cell) [82]. 3D architectures operate through physical field regulation and stress accommodation rather than purely chemical SEI programming. Their effectiveness increasingly extends to pouch‐scale demonstrations, establishing collector topology as a central design axis alongside electrolyte chemistry.

Plasma modification provides a versatile route for tailoring the electronic structure and defect landscape of collector surfaces without introducing bulk architectural complexity. Plasma treatment primarily operates by generating nitrogen‐doped carbon layers, defect‐rich interfaces, and modified surface states that enhance sodiophilicity and regulate interfacial charge transfer kinetics. These electronically tuned surfaces reduce the nucleation barrier and facilitate more uniform Na^+^ desolvation and deposition. Nitrogen‐doped carbon layers formed via plasma treatment of aluminum create conductive, chemically active interfaces that promote homogeneous sodium nucleation. In AF sodium dual‐ion configurations, such collectors have been shown to lower interfacial resistance and improve Na^+^ transfer kinetics, supporting energy densities exceeding 380 Wh kg^−1^ at moderate power densities while maintaining stability at elevated discharge rates [83]. When coupled with weakly solvating electrolytes, plasma‐modified Al@C collectors achieve average plating/stripping CE values approaching 99.9% (Na‖Cu half‐cell), and retain substantial energy density even under cryogenic conditions (−40°C) at pouch‐cell scale [21]. Plasma treatment alters the local electronic environment and defect density at the carbon‐metal interface, thereby modulating Na^+^ adsorption energy and desolvation dynamics. This distinguishes plasma engineering from purely geometric or alloy‐based strategies: rather than relying on lattice matching or porosity, it enhances nucleation homogeneity by tuning surface states and accelerating interfacial kinetics. Plasma‐modified collectors expand the operational window of AFSMBs toward high‐power and low‐temperature regimes without the mass penalties associated with bulk 3D scaffolds.

Alloy‐based interfaces regulate sodium deposition by modulating interfacial free energy, lattice compatibility, and initial SEI formation thermodynamics. Alloy coatings alter the intrinsic wetting behavior and nucleation barrier through favorable Na‐metal interactions. By introducing sodiophilic elements with lattice parameters closer to metallic Na, alloyed collectors reduce the critical nucleation overpotential and promote planar, dense deposition. Soft metal coatings such as indium (In) exemplify this principle. Compared with bare copper or aluminum, In‐coated collectors exhibit improved lattice matching and stronger Na affinity, enabling uniform growth and sustained stripping at practical cathode loadings (AF pouch) [42]. Similarly, thin intermetallic layers including Sn‐Cu and Sn‐Ni act as sodiophilic nucleation seeds, lowering interfacial energy and supporting high‐capacity AF full cells with stable cycling beyond 200 cycles [84]. High‐entropy alloy (HEA) nanoplates further extend this concept by providing a dense distribution of active nucleation sites and elastic buffering capability, facilitating extended cycling stability in AF configurations [85]. Vacancy‐rich Na–Sb–Te skeletons promote planar wetting and dense deposits; an AF NST collector||NVP cell exhibited only ≈0.23% capacity loss per cycle [50]. Beyond thermodynamic wetting control, certain alloy or catalytic interlayers actively influence early‐stage SEI chemistry. For example, Ru‐decorated copper surfaces accelerate NaPF_6_ decomposition to form ultrathin NaF‐rich interphases, coupling nucleation regulation with catalytic interphase programming [86]. Across these systems, alloy engineering reduces nucleation barriers, smooths deposition morphology, and improves capacity retention under practical areal loadings. Unlike topological 3D scaffolds that primarily redistribute ion flux, alloy interfaces operate by directly lowering the energetic cost of Na nucleation and modifying interfacial reaction pathways. This makes alloying a powerful strategy for stabilizing early‐cycle sodium deposition without the volumetric or gravimetric penalties associated with bulk porous hosts.

Chemically functional coatings provide a strategy to pre‐engineer artificial interphases that regulate ion transport and nucleation behavior before sodium deposition. Unlike alloy layers that reduce nucleation barriers through lattice matching, these coatings act as functional interlayers that homogenize ion flux, tune local dielectric environment, and guide SEI formation from the first plating event. Open‐framework coatings such as Prussian blue analogues (PBA@Cu) exemplify transport‐regulating interphases. Their ordered channels enable rapid Na^+^ diffusion while simultaneously moderating anion distribution near the surface (Figure 8f), promoting uniform plating and inorganic‐rich SEI formation [41]. Similarly, hard‐carbon‐derived interphases deposited on aluminum create low‐dielectric, Na‐affinitive surfaces that enforce homogeneous nucleation and suppress crack formation during stripping (Figure 9i) [46]. In both cases, the coating does not primarily serve as a storage host, but as a kinetic regulator of ion flux and stress evolution, enabling extended cycling and improved reversibility in AF configurations. Hybrid designs further demonstrate how thin functional layers can redistribute local pressure and stabilize interphase integrity. For example, carbon‐based nucleation layers combined with soft metal films mitigate SEI fracture and reduce dead sodium formation by smoothing interfacial stress gradients [28]. Across these systems, chemically functional coatings operate by programming the interfacial environment, balancing ion transport, nucleation thermodynamics, and mechanical compliance, without requiring bulk structural modification of the collector. This approach differs from alloying or plasma engineering: rather than altering intrinsic metal‐Na bonding or electronic surface states, functional coatings impose a controlled ion‐conduction and stress‐buffering layer that shapes early‐stage deposition dynamics. This makes artificial interphase design an attractive route for improving reversibility while maintaining compatibility with planar, scalable collector architectures.

**FIGURE 9:**
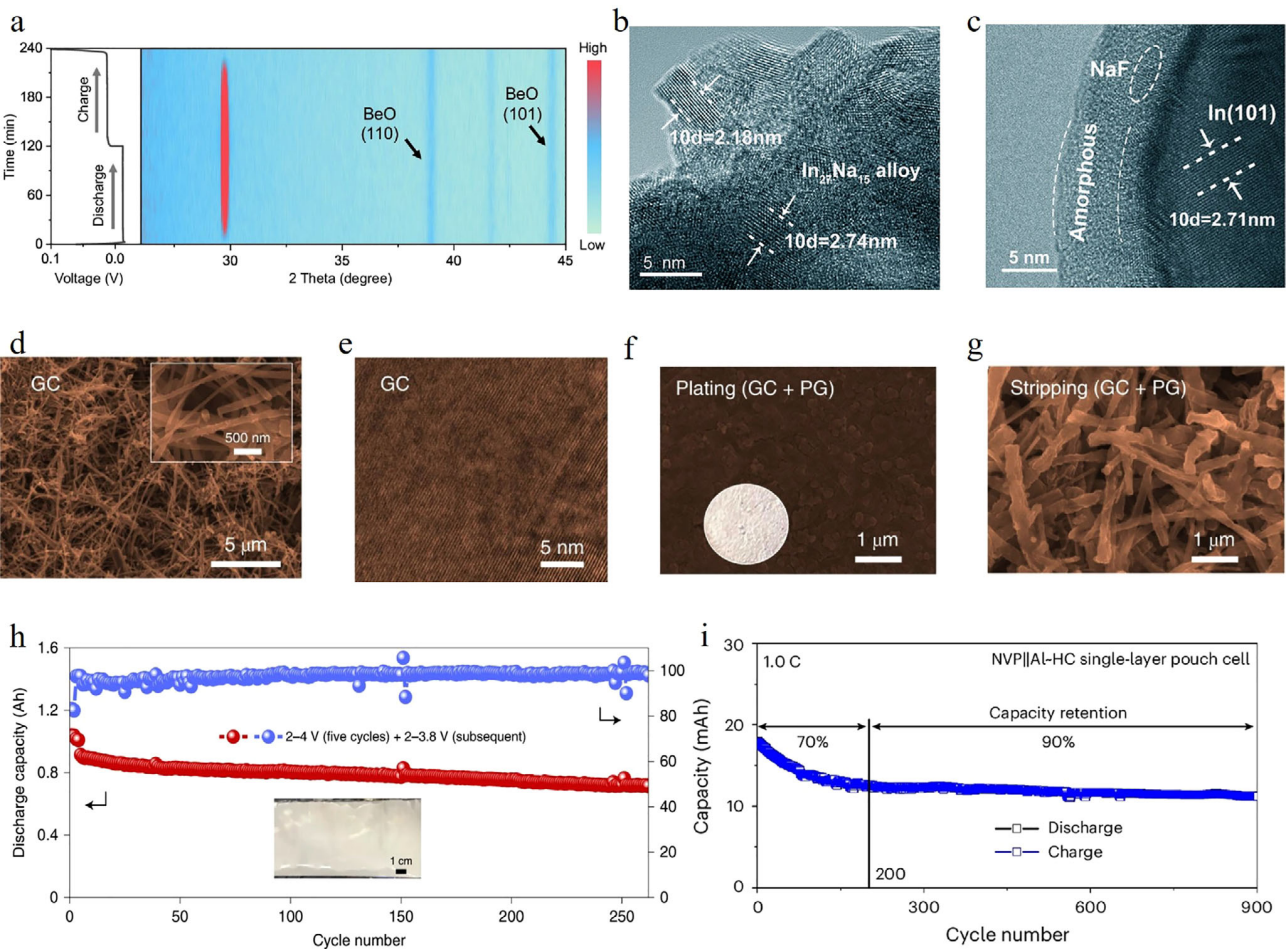
From interface design to practical validation of current collectors. (a) In situ XRD patterns demonstrating highly reversible Na plating/stripping (Adapted with permission from [66]. Copyright 2025, Springer Nature). (b, c) TEM characterization of an alloy interface showing (b) the alloy‐based sodiophilic interlayer and (c) the resulting NaF‐rich SEI enabling uniform deposition (Adapted with permission from from [42]. Copyright 2025, Wiley). (d‐g) Ex situ microscopy sequence of a graphitic‐coated collector: (d) pristine structure, (e) high‐resolution TEM of the interface. (f) dense Na plating, (g) post‐stripping surface, and (h, i) Performance in practical cell formats: (h) cycling stability of an industrial‐grade cylindrical cell using a graphitic carbon interface (Adapted with permission from [55]. Copyright 2023, Springer Nature). (i) Long‐term cycling of an anode‐less pouch cell for 900 cycles by use of hard carbon (Adapted with permission from [46]. Copyright 2025, Springer Nature).

The most advanced AFSMB systems increasingly demonstrate that collector engineering and electrolyte design cannot be optimized independently. While electrolyte chemistry defines solvation structure and decomposition hierarchy, the collector determines local electric field distribution, nucleation thermodynamics, and stress evolution. When these two elements are deliberately matched, synergistic stabilization emerges. A representative example is the combination of graphitic carbon‐coated aluminum (GC@Al) with boron‐containing electrolyte formulations, where electronically conductive carbon surfaces align with controlled interphase formation to enable efficient Na plating/stripping and energy densities exceeding 200 Wh kg^−1^ at the Ah scale (Figure 9d–h) [55]. In such systems, the collector promotes uniform nucleation, while the electrolyte ensures stable SEI/CEI chemistry; neither component alone achieves equivalent performance. Similarly, fluorinated aluminum paired with FSI‐based electrolytes, or plasma‐modified carbon‐coated aluminum combined with weakly solvating dual‐salt systems, demonstrates enhanced reversibility under lean‐electrolyte conditions. These results establish a boundary‐condition principle: electrolyte chemistry governs reaction pathways, whereas collector architecture governs where and how those reactions initiate. Properly aligning nucleation energetics, interphase composition, and ion‐flux distribution minimizes sodium loss across both early‐cycle and steady‐state regimes.

Despite substantial progress in interfacial and structural design, translating advanced collector architectures into industrially viable AFSMBs remains non‐trivial. Many high‐performing porous or nanostructured hosts rely on multistep synthesis routes that are incompatible with roll‐to‐roll manufacturing or impose significant dry‐room and cost burdens. While 3D scaffolds effectively mitigate dendrite formation and local stress accumulation, their added mass and reduced tap density can compromise gravimetric and volumetric energy density at the cell level. Similarly, surface treatments such as extensive fluorination, CVD, or ALD introduce additional processing complexity that may not scale economically. Alloy‐based or catalytic interlayers improve nucleation thermodynamics, yet long‐term durability beyond several hundred cycles at high cathode loadings remains insufficiently standardized. Furthermore, most demonstrations are conducted under laboratory‐optimized conditions that do not fully reflect lean‐electrolyte, high‐areal‐capacity constraints required for commercialization.

Progress toward industrial adoption, therefore, requires a shift from isolated material optimization to system‐level validation. Standardized testing protocols for collector‐electrolyte pairs under practical N/P ratios, E/C ratios, and areal loadings are essential. Equally important is the development of modification strategies compatible with scalable deposition techniques, such as thin‐film fluorinated carbon coatings or plasma treatments adaptable to continuous processing. Finally, techno‐economic assessments must accompany materials innovation to quantify the cost‐per‐kWh impact relative to performance gains. Without such integration, many promising collector designs risk remaining confined to laboratory‐scale validation rather than enabling commercially competitive AFSMB platforms.

### 4.3 Sodium Inventory and Crosstalk

In AFSMBs, sodium inventory and cross‐electrode crosstalk jointly govern cell‐level stability. In the absence of excess metallic Na, any irreversible loss, whether originating from cathode‐side reactions such as CEI growth, electrolyte oxidation, or transition‐metal dissolution, or from anode‐side inefficiencies, directly reduces the available sodium inventory and accelerates capacity fade. Consequently, cathode design in AF architectures must address three coupled constraints: sodium inventory retention, high‐voltage oxidative stability, and SEI‐CEI crosstalk across the cell.

A fundamental design lever involves regulating sodium inventory at the cathode. Sodium‐rich cathodes, such as Na_5_V_2_(PO_4_)_3_F_3_, and related phosphate frameworks function as internal sodium buffers that compensate for first‐cycle irreversible losses [87]. These cathodes provide additional Na^+^ during initial SEI formation, stabilizing early plating dynamics and extending cycle life under practical areal loadings (≈2.5‐3.0 mAh cm^−2^). However, their moderate operating voltage limits cell‐level energy density compared with high‐voltage layered oxides. To achieve competitive gravimetric energies, AFSMBs increasingly employ high‐voltage cathodes. Layered O3‐type NaTMO_2_ compositions and polyanionic frameworks operated at upper cut‐off voltages of 4.2‐4.5 V can theoretically raise cell‐level energy density by more than 25% relative to NVP‐based systems (Figure 10a). Yet increasing voltage simultaneously intensifies electrolyte oxidation and transition‐metal dissolution, imposing stricter interphase requirements in AF configurations.

**FIGURE 10:**
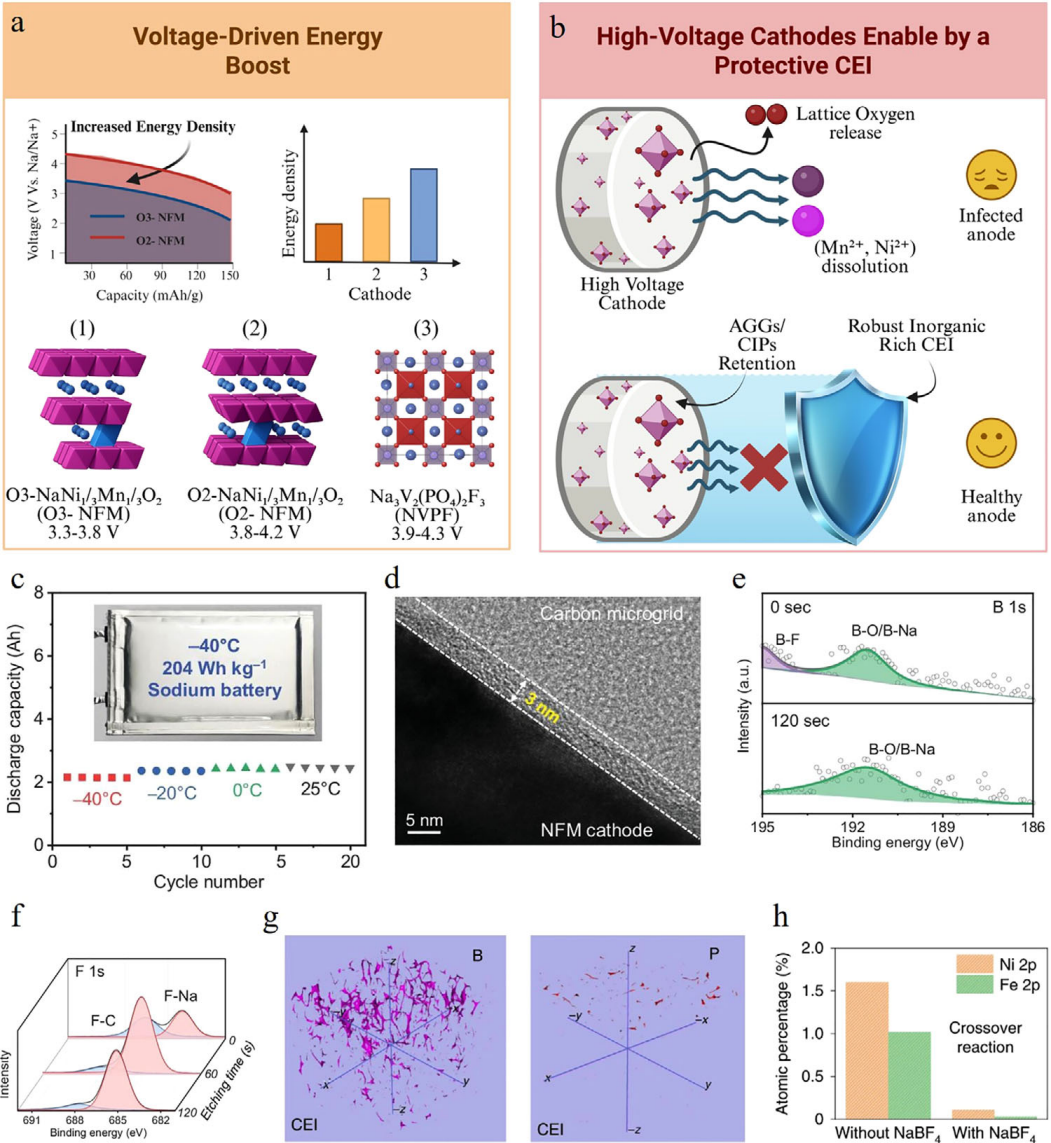
Mechanistic role of high‐voltage cathodes in enabling high‐energy, stable AFSMBs. (a) Design principles for high‐energy cathodes: voltage‐capacity profiles showing energy density gains, crystal structure considerations; (b) the role of anion‐rich solvation in forming protective cathode‐electrolyte interphases (CEIs) preventing anode poisoning and enabling stable high‐voltage operation. (c) Practical validation: temperature‐tolerant cycling performance in a pouch cell configuration achieving 204 Wh kg^−1^ at −40 °C. (d‐h) Multi‐technique characterization of the protective CEI: (d) TEM revealing an ultra‐thin (≈3 nm) CEI on NFM cathode, (e, f) XPS depth profiling showing B‐O/B‐Na and F‐Na species, (g) 3D ToF‐SIMS reconstruction illustrating stratified B^+^ and P^−^ distribution, and (h) Quantitative XPS analysis demonstrating suppressed transition metal dissolution on the Na anode. (Panels c‐f are adapted with permission from [70]. Copyright 2025, Oxford Academic, and Panels g, h are adapted with permission from [55]. Copyright 2023, Springer Nature).

At voltages exceeding ≈4.0 V vs. Na^+^/Na, layered and polyanionic cathodes trigger accelerated electrolyte oxidation, oxygen activity, and transition‐metal dissolution. In AF systems, where sodium inventory is finite, dissolved species migrate to the anode, contaminate the SEI, and exacerbate sodium loss (Figure 10b). Stabilizing high‐voltage cathodes, therefore, relies less on discovering new active materials and more on forming robust, inorganic‐rich CEIs that suppress parasitic oxidation and metal crossover. Electrolyte formulations that favor anion‐derived decomposition products have proven particularly effective in this regard. Dual‐salt systems and weakly solvating electrolytes promote the formation of NaF‐, B‐O^−^, and P‐O^−^enriched CEIs that limit surface reconstruction and suppress transition‐metal dissolution [40, 55]. Such interphase control has enabled practical pouch cells to operate up to ≈4.3 V across wide temperature ranges (−40 to 60 °C) with cell‐level energies exceeding 200 Wh kg^−1^ (Figure 10c). In this framework, cathode advancement in AFSMBs emerges primarily from electrolyte‐interface engineering rather than intrinsic changes to cathode bulk chemistry.

SEI‐CEI crosstalk represents a defining challenge unique to AF configurations. Oxidative decomposition at high‐voltage cathodes generates acidic species, reactive oxygen fragments, and dissolved transition‐metal ions. These species migrate to the anode and incorporate into the SEI, where they disrupt ionic conductivity, alter mechanical integrity, and accelerate dead sodium formation. Evidence of such cross‐electrode degradation includes NaPO_x_ dissolution, Mn^2+^ migration, and correlated gas evolution signals across both interfaces. Mitigating crosstalk requires chemically compatible interphases at both electrodes [21, 40, 55]. Electrolytes that generate similar inorganic‐rich compositions, dominated by NaF, B‐O, and P‐O species, on both CEI and SEI establish a chemically aligned interfacial environment (Figure 10d–h). By synchronizing cathode and anode interphase chemistry, these systems suppress transition‐metal poisoning and stabilize long‐term sodium cycling. Design concepts derived from Li systems remain underutilized, yet pertinent. Artificial CEIs deposited via atomic layer deposition, such as thin Al_2_O_3_ coatings, have the potential to stabilize high‐voltage cathodes in Li metal cells by mitigating oxidative degradation and metal dissolution [88, 89]. Analogous coatings on Na cathodes can fulfill a similar function. Furthermore, dissolved‐metal scavengers that chelate transition metals within the electrolyte have been effective in suppressing Mn and Ni contamination in Li‐ion systems [90]. Beyond layered oxides, several cathode families offer alternative trade‐offs between energy density and stability. NASICON‐type materials such as NVP and NaTi_2_(PO_4_)_3_ remain widely adopted owing to their structural robustness and reliable cycling under practical loadings (≈12 mg cm^−2^), albeit with moderate energy density [66, 77]. Sodium‐rich phosphates, including Na_5_V_2_(PO_4_)_3_ and NFPP, compensate for first‐cycle losses by supplying internal Na reservoirs, extending cycle life without external sodium sources [42, 87]. Conversion‐type cathodes (e.g., Cu_2‐X_Se) provide high capacities and rate capability [91], while anion‐host cathodes such as polytriphenylamine eliminate desolvation limitations by storing solvation‐free anions, enabling high power density [83]. Each chemistry reflects a different compromise among energy, stability, and sodium inventory management.

Despite diverse cathode chemistries, few studies evaluate performance under simultaneously high areal capacity, lean electrolyte, and wide‐temperature conditions representative of industrial operation. Consequently, no universally optimal cathode chemistry currently exists for AFSMBs. Instead, cathode selection must be co‐optimized with electrolyte and current collector design according to application‐specific priorities, whether energy density, power capability, or long‐term stability. System‐level integration, rather than isolated cathode innovation, is therefore essential for translating laboratory demonstrations into commercially viable AF sodium cells.

### 4.4 Solid‐State Interphase

The integration of solid‐state electrolytes (SSEs) into AFSMBs addresses a constraint that is particularly severe in AF architectures: the absence of excess sodium to compensate for interfacial losses. In liquid systems, electrolyte wetting partially mitigates contact instability, whereas in solid‐state configurations, sodium deposition occurs across rigid solid‐solid interfaces where mechanical mismatch and ionic inhomogeneity can rapidly induce void formation or short‐circuiting. Consequently, reversible Na plating in solid‐state AF cells requires simultaneous control of interfacial contact, mechanical confinement, and Na^+^ flux uniformity at the current collector boundary. Recent advances demonstrate that such control can be achieved through three complementary mechanisms: (i) mechanical densification to eliminate void‐induced hotspots, (ii) ferroelectric interlayers that homogenize electric fields, and (iii) crystallographic engineering that directs Na dissolution pathways. Representative systems, including pelletized aluminum collectors paired with sodium borohydride SSEs, textured Na_3_Zr_2_Si_2_PO_12_ ceramics, and BaTiO_3_‐based ferroelectric interlayers, have achieved Coulombic efficiencies exceeding 99.9% and nucleation overpotentials as low as ≈4 mV (Figure 11a) [30, 92, 93]. These studies collectively establish that solid‐state AF stability is governed by interfacial precision rather than bulk ionic conductivity alone.

**FIGURE 11:**
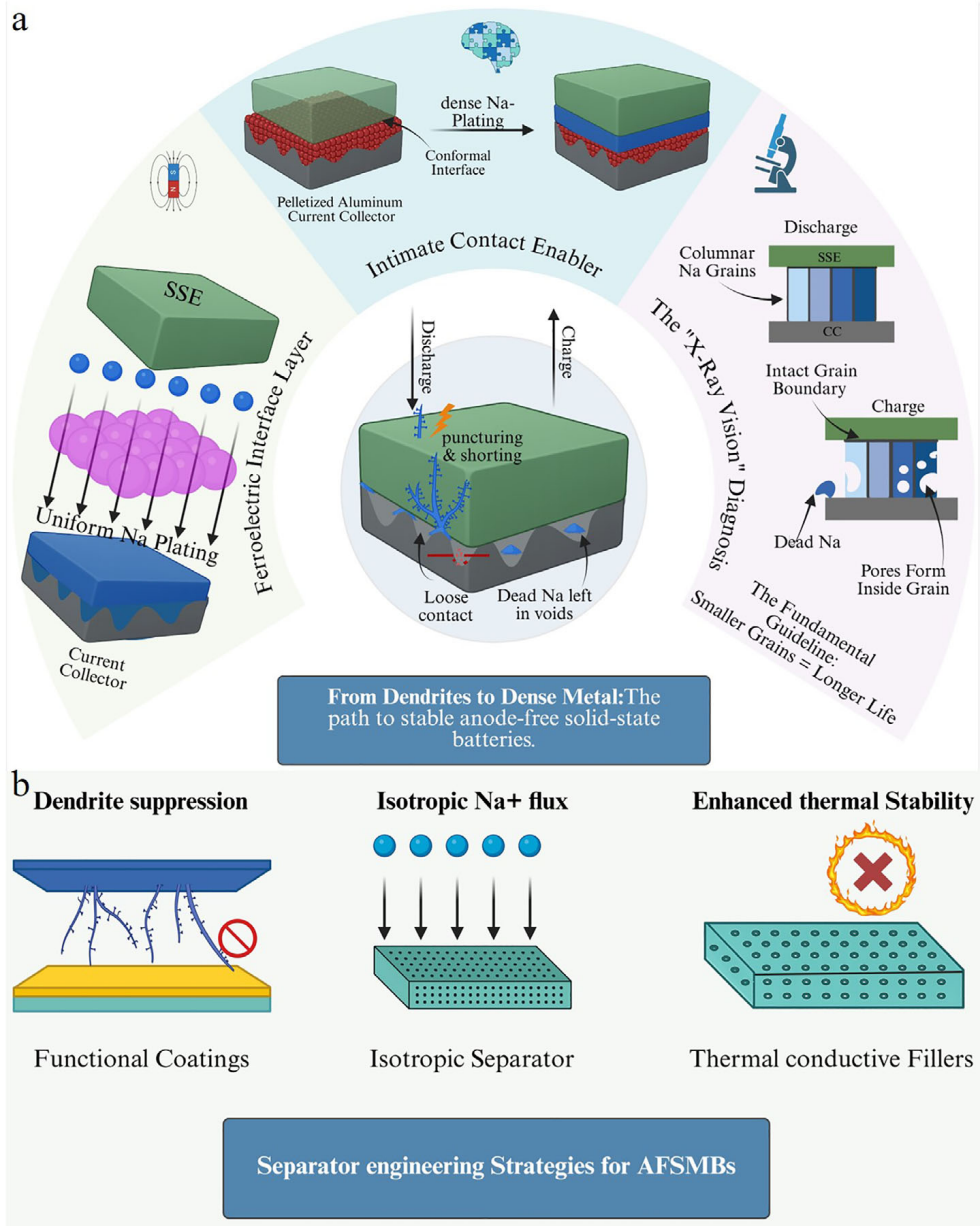
Strategies for stable sodium metal cycling in anode‐free solid‐state batteries and separator engineering for AFSMBs. (a) The inherent challenges of dendritic growth and interfacial void formation at the current collector/SEI are addressed by: a ferroelectric BaTiO_3_ composite interlayer that homogenizes Na^+^ flux via a built‐in electric field [93], a cold‐pressed, dense aluminium powder current collector that ensures intimate interfacial contact [30], and microstructural analysis revealing that pore formation during stripping occurs preferentially within grains, not at grain boundaries, providing a critical design principle for microstructural control [92], enabling the dense, reversible Na deposition essential for high‐energy‐density storage. (b) Isotropic separators homogenize Na^+^ flux, the only strategy proven in AFSMBs [94]. Functional coatings block dendrites, a concept validated in SMBs [60]. Thermally conductive fillers enhance safety, as demonstrated in Li‐based systems [95]. These strategies represent promising future directions for AFSMBs.

A central challenge in solid‐state AFSMBs lies in reconciling high ionic conductivity with interfacial stability. Sulfide‐based SSEs can reach conductivities exceeding 30 mS cm^−1^ at room temperature [96], whereas halide‐ or polymer‐based systems often exhibit conductivities orders of magnitude lower [97]. However, high bulk conductivity alone does not guarantee uniform Na^+^ transport across solid‐solid junctions. Contact resistance, interfacial void formation, and grain‐boundary anisotropy frequently dominate plating behavior. Recent strategies, therefore, prioritize interfacial engineering alongside conductivity optimization. Mechanical densification of aluminum collectors against borohydride electrolytes reduces void‐induced current hotspots by ensuring intimate contact. Ferroelectric BaTiO_3_ interlayers introduce built‐in polarization fields that homogenize Na^+^ flux and reduce local overpotential. Meanwhile, crystallographic texture control in NASICON‐type ceramics reveals that grain orientation critically influences Na stripping uniformity, with specific grain‐boundary alignments suppressing pore formation during cycling. These findings highlight that, in solid‐state AFSMBs, ionic transport must be considered jointly with mechanical coherence and microstructural anisotropy.

From a functional perspective, solid‐state AFSMBs offer three inherent advantages: elimination of flammable solvents, mechanical resistance to dendrite penetration, and compatibility with solvent‐free processing routes. However, the transition from laboratory demonstration to scalable implementation remains challenging. Rigid SSEs are prone to contact loss under repeated Na plating and stripping, particularly in the absence of self‐healing liquid wetting. Interfacial resistance accumulates as voids form during stripping, and scalable fabrication of dense, defect‐free SSE layers with controlled microstructure remains technically demanding. Future progress will likely rely on hybrid architectures that combine the high conductivity of inorganic sulfides with the mechanical compliance of polymer electrolytes, alongside thin interfacial coatings that relieve stress and maintain ionic continuity. Advanced operando and cryogenic characterization techniques will be essential for resolving the evolution of buried interfaces in real time. Importantly, insights gained from AF solid‐state systems extend beyond sodium chemistry, reinforcing a broader principle: in solid‐state batteries, interfacial precision and mechanical coherence dictate reversibility more strongly than bulk transport metrics alone.

### 4.5 Ion Transport Regulation

Regulating Na^+^ transport is critical for stabilizing deposition in AFSMBs, where sodium‐limited architectures are highly sensitive to flux inhomogeneity. In contrast to conventional sodium‐metal cells, where excess Na can partially buffer interfacial inhomogeneities, AF configurations are highly sensitive to localized ionic current amplification. Even minor flux heterogeneity can initiate dendritic nucleation and accelerate irreversible sodium loss. Wu et al. demonstrated that commercial polyolefin separators with anisotropic pore structures create localized Na^+^‐flux hotspots, exacerbating heterogeneous deposition and dead sodium formation under AF conditions [94]. By contrast, an isotropic separator fabricated via thermally induced phase separation achieved multiscale in‐plane pore uniformity, ensuring spatially homogeneous Na^+^ transport. Surface functionalization further improved wettability and Na^+^ affinity, reducing desodiation overpotential by ≈40% relative to Celgard membranes. Under AF cycling, cells employing this isotropic separator retained ≈85% capacity after 200 cycles, accompanied by a substantial reduction in dead sodium accumulation.

The separator functions as an ionic current redistributor rather than a passive mechanical barrier. While 3D collectors mitigate nucleation heterogeneity and electrolytes regulate interphase chemistry, the separator governs the spatial distribution of Na^+^ flux arriving at the collector surface. This establishes separator engineering as a complementary and modular stabilization lever that is compatible with both liquid and solid‐state AF systems. Beyond isotropic pore design, Figure 11b outlines emerging directions inspired by related chemistries, including dendrite‐suppressive surface coatings and thermally conductive fillers that enhance heat dissipation. Although these approaches remain underexplored in AF sodium systems, they suggest a broader design framework in which ionic flux control, dendrite mitigation, and intrinsic thermal management are integrated within a scalable separator architecture.

The strategies surveyed above are compiled in Table 2 against the parameters that determine AF viability, including N/P ratio, areal capacity, electrolyte‐to‐capacity ratio, and cell format. The six strategy classes are not interchangeable, and their relative standing depends on which constraint is binding. Electrolyte design is the most readily scaled, since it requires no change to electrode manufacture and is compatible with existing coating lines, and it is the only class represented among systems meeting lean‐electrolyte criteria; its limitation is that solvation‐derived gains reported in flooded coin cells do not necessarily persist at low E/C. Coatings and alloy interlayers offer the largest reductions in nucleation overpotential per unit added mass, but introduce a vacuum deposition or wet‐chemical step whose cost scales with electrode area. Three‐dimensional architectures give the most uniform current distribution but carry an inactive‐mass penalty that directly offsets the energy‐density advantage motivating AF design, and their high surface area increases the area available for chemically driven inventory loss. Crystallographic and single‐crystal approaches are mechanistically informative but presently confined to model substrates. Solid‐state configurations remove the electrolyte inventory problem entirely at the cost of stack pressure requirements incompatible with current pouch formats. Assessed against practical metrics rather than cycle life alone (Table 2), and against manufacturing readiness (Table 4), the reported advances narrow considerably, which motivates the system‐level analysis that follows.

**TABLE 2:** Practical performance metrics of representative AFSMB strategies.

Reference	Strategy class	System / key modification	Cathode (loading, mg cm^−2^)	N/P	ICE (%)	Avg CE (%)	Areal capacity (mAh cm^−2^)	E/C (g Ah^−1^)	Cycles/retention (%)	Cell format	Energy density (Wh kg^−1^) [basis]
[55]	CC + electrolyte	Graphitic carbon on Al with borate/phosphate (BPG) electrolyte	Na[Cu_1/9_Ni_2/9_Fe_1/3_Mn =]O_2_ (>15)	0	∼92	NR	∼2 (coin/pouch); ∼3 (cylindrical)	2	260 / 84	Coin, pouch, cylindrical (1 Ah)	205 (2‐4 V); 180 (2‐3.8 V) [cell]
[46]	CC	Hard‐carbon‐derived interphase on Al	NFPP (∼20)	>0.5	92.5	99.94	2 (2.28Ah pouch) single face	13(pouch), 2.2 (2.28 Ah)	900 / 90 (pouch)	Coin, pouch (2.8 Ah)	203 [cell]
[66]	CC	F‐doped mesoporous carbon nanofibre (FMCNF)	NVP (20); NVOPF (16)	0	NR	99.95	2.34 [calc.]	NR	200 / 90 (pouch)	Coin, pouch	322 (NVP); 383 (NVOPF) [active]
[21]	CC	Plasma‐treated N‐doped carbon on Al + weakly solvating dual‐salt	NVP (15)	0	NR	99	1.5 [calc.]	7	50 / 93.8 (pouch)	Coin, pouch (∼1.79 Ah)	110 [cell]
[44]	CC	MOF‐derived fluorinated carbon thin film (FCTF)	NVP (∼2.5 (coin); ∼7.1 (pouch))	0 (coin); 1.5 (pouch)	90.5	99.7	0.83	NR	400 (coin); 300 / 94.7 (pouch)	Coin, pouch (16 cm^2^)	325 [active]
[78]	CC	In situ grown Cu_3_P nanowires on Cu	NVP (13.6)	0	81.1	99.8	NR	NR	170 (pouch)	Coin, pouch	NR
[42]	CC	Indium coating on Cu (lattice matching)	NFPP (≥18)	0	90.87	NR	NR	NR	320 at 2C / 78.3	Pouch	NR
[27]	CC	Zn‐Nx doped carbon nanofibre (Zn‐CNF)	NVP (7.97)	0	95.8	99.94	NR	NR	120 / 80.4	Coin	374 [cathode basis]
[75]	CC	N,O dual‐doped carbon spheres	NFM (7.52, AF)	0 (AF); 2.18 (anode‐less)	95.45 (AF)	99.87	NR	NR	100 / 85.5 (AF)	Coin (16 cm2 pouch demo)	337.9 (AF) [active]
[15]	CC	Strain‐engineered single‐crystal Al(100)	NVP (∼1.5)	0	98.5	99.9	0.18	NR	100 / 68	Coin	NR
[86]	CC	Ru/NC catalytic layer on Cu	NVP (3)	0	90.75	∼100	NR	NR	100 / 98.1	Coin	NR
[41]	CC	In situ Prussian blue analogue interlayer on Cu	NVP (∼3)	0	NR	99.14	NR	NR	300 / 75.3	Coin	NR
[85]	CC	High‐entropy alloy (NbMoTaWV) nanolayer on Al	NR	0	NR	99.5	NR	NR	300 / 96.5	Coin	NR
[77]	CC	Bi nanosheet arrays on Cu mesh	NTP	0 (AF); 1 (anode‐less)	NR	99.8	NR	NR	267 / 93.2	Coin	NR
[84]	CC	Sn‐Cu alloy layer on Al	NVP (7.6)	0 (AF); 1 (anode‐less)	NR	99.95	NR	NR	200 / 90	Coin	NR
[22]	Electrolyte	Weakly solvating anion‐stabilized electrolyte	NFM (22)	0	NR	99.89	2.64	4	100 / 82.3 (coin); 50 / 80 (pouch)	Coin, multilayer pouch	153.3 [cell]
[40]	Electrolyte	Sole‐solvent high‐entropy electrolyte (D2 + BPG)	NFM (>20)	0	NR	NR	2.6	NR	100 / 83.1 (25 C, pouch)	Pouch (Ah‐level)	209 [cell]
[70]	Electrolyte	Association‐dissociation equilibrium regulation (NaBF4 in G2/G4)	NFM (21)	0	NR	99.9	NR	NR	100 / 87 (‐40 C)	Pouch (Ah‐level)	204 [cell]; 443 [cathode+anode]
[48]	Electrolyte	Dipole‐dipole interaction regulation	NVP (>10)	0	NR	99.95	∼5	2.1 (0.5C, 1.1 Ah); 1.9 (1C, 0.82 Ah)	480 / 80–83 (coin); 700 / 81 (pouch)	Coin, pouch	150 [cell]
[23]	Electrolyte	3A zeolite molecular‐sieve film (AGG‐dominated)	NNCFM	0	NR	99.91	∼3 (estimated)	NR	250 / 73.1	Coin	369 (NNCFM); 372 (NVPF) [active]
[71]	Electrolyte	Nano‐silica suspension (anion scavenging)	NVP (4)	0	77.75	99.5	NR	NR	50 / 75	Coin	326.7 [active]
[87]	Cathode	Sodium‐rich NASICON cathode as internal Na reservoir	Na‐rich NASICON (7)	0	90	99.8	1.5	NR	300 / 95 (3.0‐3.8 V)	Coin, pouch	400; 320 after 300 cycles [active]
[94]	Separator	In‐plane isotropic PE separator	NVP (6.0); NNMFO (7.0; 13.5)	0	93.2 (NVP); 86.1 (NNMFO)	99.5	0.89‐1.7	NR	400 / 83.8 at 4C (NVP)	Coin	346 (NVP); 356 (NNMFO) [cathode basis]
[30]	Solid‐state	Na borohydride solid electrolyte + stack pressure	NaCrO_2_	0	93	99.96	7	NR	400 / 70	Coin	NR

*Note*. Twenty‐four systems spanning current‐collector (CC), electrolyte, cathode, separator, and solid‐state approaches, compiled against the parameters that determine AF viability. N/P denotes the negative‐to‐positive capacity ratio, with entries labelled AF (N/P = 0, bare collector) or anode‐less (pre‐sodiated or N/P > 0) following the definitions in Section 2.2. NR indicates a parameter not reported in the source. Energy densities are annotated with their calculation basis, which can differ by up to a factor of two among cell‐level, active‐material, and cathode‐mass values, and must not be compared across bases. Electrolyte‐to‐capacity ratio (E/C) is reported in only five of the 24 systems, and only entry 19 satisfies both of the practical thresholds of E/C ≤ 3 g Ah^−^^1^ and areal capacity ≥ 3 mAh cm^−^^2^; entry 1 approaches them in a cylindrical format. Entry 2 reports 13 g Ah^−^^1^ in a single‐layer pouch against 2.2 g Ah^−^^1^ in a 2.28 Ah cell of the same chemistry, illustrating that E/C is set by cell format rather than by chemistry. Complementary to Table 4, which assesses the same literature for manufacturing scalability and technology readiness.

## 5 Scaling anode‐free sodium metal batteries

### 5.1 Lab‐to‐Fab Gap

While the interfacial strategies discussed in Section 4 effectively regulate nucleation, interphase chemistry, and sodium deposition at the material level, their translation to practical cell formats reveals additional constraints that are not captured in laboratory‐scale evaluations. Scaling exposes system‐level limitations, where electrochemical performance becomes inseparable from mechanical, architectural, and manufacturing parameters. Scaling constraints and performance gaps are summarized in Figure 12. At this stage, the governing physics shifts from interface‐controlled behavior to system‐level coupling, where electrochemical, mechanical, and transport processes become intrinsically linked. Coin‐cell data often report a CE exceeding 99.9% and stability over 100–300 cycles; however, these results are obtained under conditions that are significantly different from production environments, typically involving 30–50 µL of electrolyte (>10 g Ah^−1^), uncontrolled pressure, and small active areas that obscure inventory and mechanical constraints. Industrial formats necessitate an areal loading of ≥3 mAh cm^−2^, electrolyte usage of ≤3 g Ah^−1^, and a uniform stack pressure of 0.1‐0.4 MPa to maintain contact without deforming the separators. Under these conditions, a chemistry that maintains 99.9% CE over 200 cycles in coin cells [84] often falls below 99% when scaled to lean‐electrolyte pouches owing to inhomogeneity in the Na^+^ flux and rapid consumption of the SEI (Figure 12a). The industrial targets used in Figure 12a are not arbitrary cut‐offs but fall into three provenance classes, listed in Table 3. Two are derived within this Review from the sodium inventory constraint: the minimum average Coulombic efficiency follows from the retention relation of Section 2.3, and the cycle‐life target is the corresponding commercial requirement against which that efficiency is evaluated. Three are format constraints reported for industrial cell assembly rather than performance aspirations, namely areal loading, electrolyte‐to‐capacity ratio, and the stack pressure compatible with separator integrity. The remaining target, cell‐level specific energy, is referenced to commercial sodium‐ion cell specifications, since an AFSMB that does not exceed them has no application case. Each metric in Figure 12a is normalized to its target value, so a score of 100 denotes attainment rather than a relative ranking.

**FIGURE 12:**
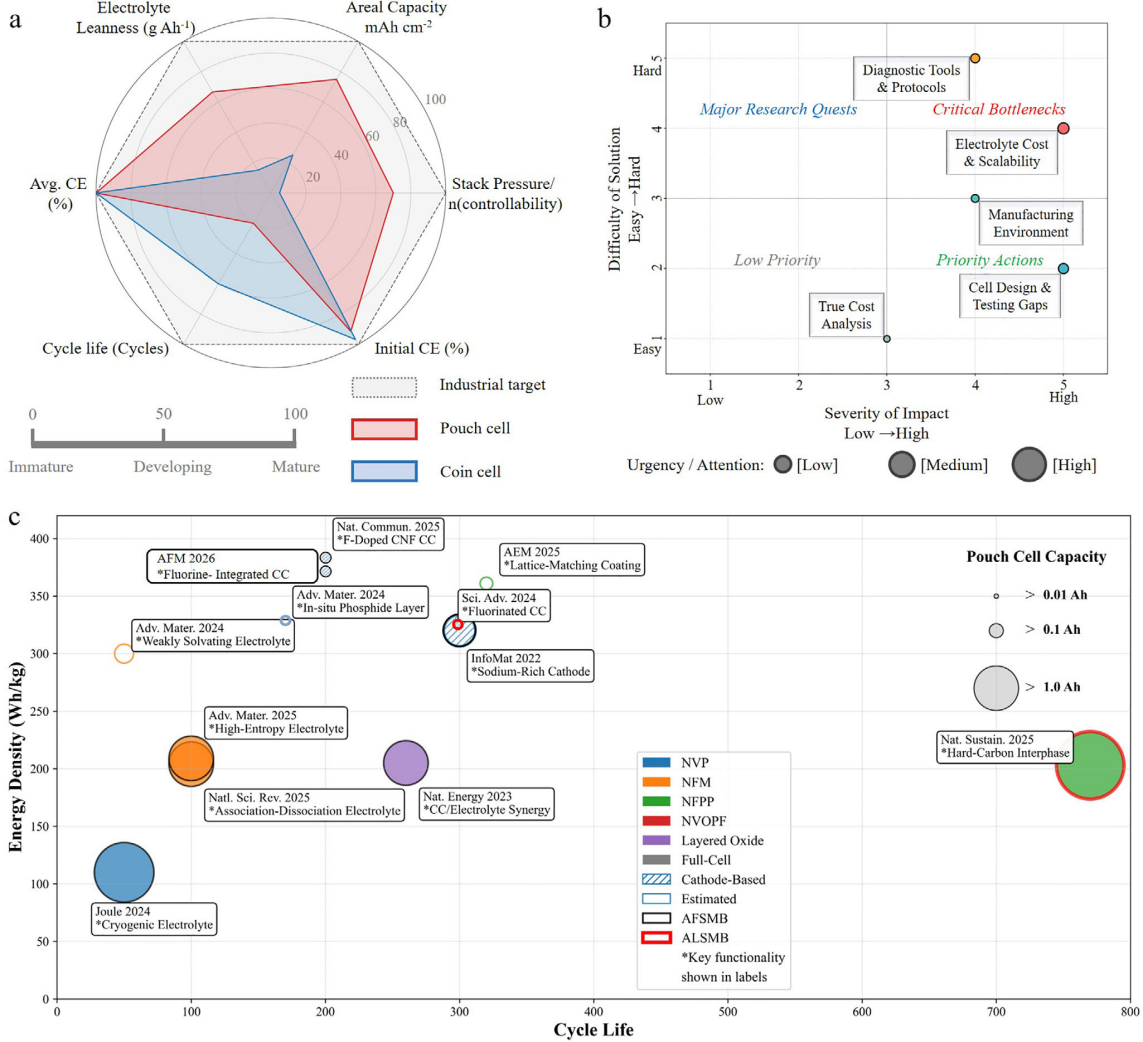
Scaling and commercialization outlook for anode‐free sodium‐metal batteries. (a) Multi‐parameter radar chart comparing lab‐scale coin cells, state‐of‐the‐art pouch cells, and industrial targets across six normalized performance metrics (100 = industrial benchmark). The gap between laboratory and practical conditions highlights the impact of uncontrolled pressure, high E/C, and low areal capacity. (b) Commercialization risk matrix showing the relative difficulty (y‐axis) and impact (x‐axis) of major AFSMB roadblocks. Scores derive from literature consensus, techno‐economic models, and supply‐chain data. (c) Performance landscape of Na‐metal pouch cells in anode‐free (solid outlines, N/p = 0) and anode‐less (red outlines, N/P ≈ 1.5) configurations. Bubble area corresponds to pouch‐cell capacity; color denotes cathode chemistry; fill style distinguishes full‐cell and cathode‐based energy densities. Current practical performance clusters near 200 Wh kg^−1^, with anode‐less cells showing superior cycle life. Abbreviations: NVP, Na_3_V_2_(PO_4_)_3_; NFM, NaNi_x_Fe_y_MnzO_2_; NFPP, Na_4_Fe_2.9_(PO_4_)_2_P_2_O_7_; NVOPF, Na_3_(VO) _2_ (PO_4_)_2_F.

**TABLE 3:** Provenance of the industrial benchmark values used in Figure 12a. Targets fall into three classes: Derived within this Review from the sodium inventory constraint, reported constraints of industrial cell assembly, and commercial sodium‐ion cell specifications. Each axis in Figure 12a is normalized to its target, so 100 denotes attainment rather than relative ranking.

Metric	Target	Basis	Source class
Average CE	≥99.96%	Derived from R = ICE × CE^(n−1)^ for 80% retention over 500 cycles (Table 1)	Derived in this Review
Cycle life	≥500 cycles	Commercial requirement against which CE is evaluated	[48]
Areal capacity	≥3 mAh cm^−2^	Cathode loading required for competitive cell‐level energy	[99]
E/C ratio	≤3 g Ah^−1^	Electrolyte inventory in industrial‐format cells	[55]
Stack pressure	0.1‐0.4 MPa	Contact maintenance without separator deformation	[98]
Specific energy	≥200 Wh kg^−1^ (cell)	Commercial Na‐ion cell specification	[70]

Electrolyte starvation and stack pressure optimization are emerging as dominant control parameters for scaling AFSMBs. Moderate compression densifies sodium deposits and suppresses void formation, whereas excessive or uneven pressure induces interfacial delamination and shorting. Xie et al. directly examined the evolution of sodium morphology under different compression levels and demonstrated that controlled mechanical confinement yields compact sodium plating and prolonged reversibility [42]. Conversely, complementary work reported that AF Na||NFM pouch cells sustained over 260 cycles at an E/C of approximately 2 g Ah^−1^ without applying external pressure, suggesting that optimal electrolyte‐collector coupling can partially offset the mechanical constraints [55]. Ruan et al. reported a 2.28 Ah aluminum‐hard‐carbon anode‐less cell with 203 Wh kg^−1^ (311 Wh L^−1^) and 95.2% capacity retention over 180 cycles at an E/C of approximately 2.2 g Ah^−1^, confirming that careful mechanical and electrolyte control can deliver Ah‐scale stability [46]. These results imply the existence of a critical pressure window, likely within 0.1‐0.4 MPa [98], where the Na^+^ flux, SEI stability, and gas evolution remain balanced, a factor that warrants systematic quantification in future industrial prototypes.

Operating with lean electrolytes reveals degradation pathways that are not apparent in flooded conditions. The hydrolysis of NaPF_6_, resulting in the formation of PF_5_ and HF, or the breakdown of solvents, leads to pouch swelling when the electrolyte volume decreases below approximately 10 µL per cell [52]. For example, recent demonstrations achieved 1.1 Ah C@Al||NVP pouch cells utilizing a fluorinated ether electrolyte (2.1 g Ah^−1^), which maintained 700 cycles with 81% capacity retention and 99.75% CE [48]. Additionally, parallel studies demonstrated that WSAS electrolytes facilitate stable operation at ‐30°C with an E/C of approximately 4 g Ah^−1^, underscoring the combined significance of solvation chemistry and thermal management [22].

### 5.2 Manufacturing Constraints

Economic and environmental limitations represent a primary barrier to the practical deployment of AFSMBs. While sodium itself is inexpensive, the supporting chemical ecosystem is not. Fluorinated salts such as NaFSI and NaTFSI are typically priced at five to six times that of NaPF_6_ and require high‐purity ether solvents or additives such as FEC, increasing electrolyte costs to approximately US $12–15 kg^−1^, approaching those of lithium‐ion systems [100, 101]. As a result, the combined cost of salts, solvents, and interfacial coatings elevates projected cell manufacturing expenses to $80–120 kWh^−1^, diminishing the anticipated cost advantage of sodium‐based chemistries. In parallel, sodium's extreme moisture sensitivity imposes stringent processing requirements. Dew points of ≤ ‐60°C are typically required to prevent parasitic reactions, leading to dry‐room capital costs that are 50–100% higher than those of conventional Li‐ion production lines [102]. Despite this, fewer than 5% of AFSMB studies report humidity tolerance or storage stability, even though trace levels of H_2_O can trigger HF formation, accelerate electrolyte decomposition, and induce catastrophic SEI failure.

Beyond cost and environment, a fundamental mismatch exists between laboratory cell designs and industrial manufacturing constraints. Academic demonstrations often employ thick (>20 µm) current collectors, excess electrolyte (50–100 µL), and uncontrolled stack pressure, whereas practical cells require ultrathin collectors (≤10 µm), lean electrolyte conditions (≤3 g Ah^−1^), and controlled compression (0.1–0.4 MPa). Under these constraints, chemistries that perform well in coin cells frequently exhibit a 1%–2% drop in CE due to edge plating, non‐uniform current distribution, and tab‐induced corrosion [42, 55]. Although seemingly minor, this decline, from 99.9% to ≈98%, is sufficient to exhaust the sodium inventory within ≈100 cycles, highlighting the extreme sensitivity of AF systems to small efficiency losses. Scaling is therefore limited not by any single material parameter but by the coupling between them. A semi‐quantitative commercialization‐risk matrix (Figure 12b) captures this transition, identifying electrolyte scalability and dry‐room processing as dominant bottlenecks, while diagnostic standardization and techno‐economic modeling remain underdeveloped but necessary components of system validation.

Attributing this penalty requires separating three mechanisms that act simultaneously but scale differently, and which can therefore be distinguished by their signatures rather than by their magnitude alone. Electrolyte starvation scales with the electrolyte‐to‐capacity ratio and with cumulative cycled capacity, produces a gradual impedance rise followed by accelerated rollover, and is distributed across the electrode area. Edge plating is geometric: it scales with the perimeter‐to‐area ratio and with cathode‐anode overhang, localizes at the perimeter and tab regions, and diminishes in relative terms as cell area increases. Stack‐pressure non‐uniformity is mechanical and is distributed through the stack rather than across it, producing layer‐to‐layer variation in contact and void closure that is most pronounced in the interior layers of multilayer assemblies and is sensitive to rate.

These signatures are separable in principle by holding two variables fixed while varying the third: E/C at constant fixture pressure and electrode geometry; perimeter‐to‐area ratio and overhang at constant area, pressure, and E/C; and applied pressure and layer count at constant geometry and E/C, with post‐mortem sodium inventory (Equation 1) mapped spatially and by layer. Such controlled comparisons are not yet available in the AFSMB literature, and their absence is the principal obstacle to attributing the pouch penalty quantitatively. The available data are nonetheless indicative. Electrolyte starvation appears to be the dominant format‐dependent variable, since the same hard‐carbon interphase chemistry requires 13 g Ah^−1^ in a single‐layer pouch but 2.2 g Ah^−1^ in a 2.28 Ah cell (Table 2), a sixfold difference arising from format rather than chemistry. Pressure effects are expected to be unusually significant for sodium specifically: at 298 K, Na sits at a homologous temperature of *T*/*T*_m_ ≈ 0.80, against ≈0.66 for lithium, so creep‐mediated deformation is active at room temperature and modest applied pressures can close voids and restore contact to otherwise disconnected deposits [103, 104]. This provides a mechanistic basis for the narrow effective pressure window reported for AFSMBs and indicates that pressure and electrolyte starvation are not independent, since compression alters both interfacial contact and electrolyte distribution within the stack [98].

Bridging the lab‐to‐fabrication gap, therefore, requires manufacturing‐aware design and standardized evaluation protocols. Key criteria include electrolyte usage of ≤3 g Ah^−1^, areal capacities of ≥3 mAh cm^−2^, controlled stack pressure, and validation in ≥1 Ah pouch cells under realistic dry‐room conditions. Measured against these criteria, the literature is sparser than aggregate performance figures suggest (Table 2). Of twenty‐four representative systems, only five report the electrolyte‐to‐capacity ratio at all, and of these, three operate at 4–13 g Ah^−1^. A single system satisfies both the ≤3 g Ah^−1^ and ≥3 mAh cm^−2^ thresholds simultaneously. Reported Coulombic efficiencies of 99.9% and above are therefore obtained almost exclusively under electrolyte‐flooded conditions, at areal capacities below 3 mAh cm^−2^, or without the electrolyte loading being stated, which limits what can be inferred about behavior at practical loading.

Salt selection is a first‐order cost determinant, though the available price data must be read with care. Laboratory‐grade catalogue prices normalize to approximately $3800–4800 per kg for NaPF_6_, $4400 per kg for NaFSI at matched supplier and pack size, and $27 000 per kg for NaTFSI, against $61–252 per kg for NaBF_4_ [105]. These figures serve only for relative comparison and do not represent battery‐grade costs at production scale, where pack‐size effects dominate: NaBF_4_ alone falls fourfold between 500 g and 10 kg quantities from the same supplier. That NaBF_4_ is the only salt of the four offered in kilogram quantities is itself a more reliable indicator of production maturity than unit price.

Cost ranking alone does not determine suitability. NaPF_6_ remains the incumbent and the only salt produced at battery scale for sodium systems, but hydrolyses to HF and POF_3_ on contact with trace water, which sets the dry‐room specification discussed below. NaBF_4_ is the cheapest and most thermally stable but has lower dissociation and ionic conductivity in common solvents, and is therefore generally reported in mixed‐salt or hybrid‐solvent formulations rather than as a sole salt. Imide salts carry a modest cost premium over NaPF_6_ rather than a prohibitive one, but impose a formulation constraint specific to AF architectures: NaFSI in propylene carbonate induces aluminium oxidative dissolution above 3.6 V at 20 °C and 3.5 V at 55 °C, although the same salt in a pyrrolidinium‐FSI ionic liquid shows no corrosion to 5 V [106]. Since AF cells plate directly onto bare aluminium and several reported systems operate to 4.3 V, FSI‐based formulations are constrained by solvent choice rather than excluded outright. Electrolyte cost also scales with the E/C ratio, so salt price and electrolyte leanness must be optimized together: a lean imide formulation at 3 g Ah^−1^ may cost no more per Ah than a flooded NaPF_6_ one at 10 g Ah^−1^.

Dry‐room expenditure is the second cost center and is specific to reservoir‐free architectures. Plated sodium is more reactive toward residual moisture than hard carbon, so AF assembly requires a dew point of approximately ‐50 to ‐60 °C, against ‐40 °C typical for sodium‐ion lines. Dehumidification capital and energy costs rise steeply as dew point falls, so this is a distinct capital commitment rather than a marginal addition to an existing sodium‐ion line. Techno‐economic analysis incorporating both terms, alongside recycling, is necessary before cell‐level cost parity with sodium‐ion can be claimed. Achieving target energy densities approaching ≈300 Wh kg^−1^ will require engineering solutions compatible with gigafactory constraints rather than optimization under coin‐cell conditions. To contextualize individual material strategies within this industrial framework, Table 4 summarizes representative AFSMB systems in terms of electrochemical performance, scalability, and technology readiness level (TRL). Notably, few strategies reach TRL 5, underscoring the early‐stage maturity of the field despite promising laboratory‐scale results.

**TABLE 4:** Performance‐scalability‐technology‐readiness assessment of representative AFSMB strategies discussed in this review and used to construct the matrix in Figure 16a. TRL scale: 2 (fundamental research), 3 (proof‐of‐concept), 4 (lab‐scale validation), 5 (prototype demonstration). Only 3 of 36 strategies (8%) reach TRL 5, highlighting the early‐stage nature of AFSMB development despite promising academic results. Performance scores (0‐5) evaluate cycle life, rate capability, and cell format; scalability scores (0‐5) assess manufacturing feasibility, cost, and material availability. Reference numbers correspond to citations in the main text. Categories: Current collector (CC), Elec. (electrolyte), SSE (solid‐state electrolyte), Separ. (separator).

Reference #	Category	Strategy	Cycle life (cell type)	Rate (C)	Scalability assessment	Performance score	Scalability score	TRL
[107]	CC	The porous Al architecture suppresses dendrites and stress by uniformizing ion flux and lowering current density.	100 (Pouch)	—	Multi‐step process: electrodeposition + cold‐pressing + annealing + physical separation + Zn evaporation + Energy intensive ☒ Zn recycling—environmentally favorable☑	3	1.5	4
[41]	CC	In situ growth of a Prussian Blue Analogue as a rapid Na^+^ diffusion interlayer @Cu	300 (Coin)	5C	Simple, low‐cost, and scalable method. ☑ Also works on Al and Stainless Steel (SS) ☑	3	4	3
[66]	CC	FMCNF structure for electronic modulation	200 (Pouch)	0.3C	Complex multistep preparation limits practical application☒	3.5	1	4
[15]	CC	A strain‐engineered Al (100) single‐crystal CC to enable uniform Na deposition	100 (Pouch)	17C	Calcination of Al foil ☑ nonpractical mass loading (1.5 mg/cm) ☒ Low absolute capacity (68 mAh g^−1^) ☒	4.5	3.5	4
[86]	CC	Ru/NC catalyst accelerates NaPF_6_ decomposition to form a NaF‐rich SEI	100 (Coin)	0.5C	Ruthenium, a precious metal, and Complex synthesis☒	1.5	2	3
[75]	CC	N&O doped carbon sphere, enhancing sodophilicity and interfacial stability.	100 (Coin)	0.45C	Complex synthesis, Long processing time☒ Energy intensive☒ Carbon‐based material☑	1.5	1.5	3
[27]	CC	Electronic structure regulation of 3D CCs using Zn‐N_X_ active sites to enhance Na affinity and stabilize the SEI.	120 (Coin)	0.5C	Multiple complex steps—MOF synthesis + electrospinning + carbonization(800°C+)☒ Carbon‐based material☑	2	1	3
[42]	CC	Indium coating @ Cu foil to improve crystal matching and lattice force with Na.	320 (Pouch)	2C	Magnetron sputtering and rare metals are expensive + energy intensive ☒ Single‐layer coating☑	4.5	2	4
[84]	CC	Ultrathin Sn‐Cu/Sn‐Ni intermetallic coatings on Al foil via magnetron sputtering to enable uniform Nucleation.	200 (Coin)	1C	Magnetron CO‐sputtering☒ Multiple targets—Sn, Sn‐Ni, Sn‐Cu ☒ Thin layer (20 nm) ☑	2.5	1.5	3
[46]	CC	Hard‐carbon‐derived interphase @ Al CC	180 (2.28Ah Pouch)	0.2C	Industrial format cell☑ Lean electrolyte (2.2 g Ah^−1^)☑ Low N/P‐Not truly AF☒	4	3	5
[81]	CC	SnO_2_ nanoparticle anchoring, mesoporous carbon framework design	200 (Coin)	0.9C	complex multi‐step synthesis☒ expensive precursors☒	2.5	1	3
[85]	CC	A high‐entropy alloy (NbMoTaWV) nanolayer is sputtered @ Al to enhance sodiopthicity.	300 (Coin)	0.5C	Five different refractory metals (Nb, Mo, Ta, W, V) are expensive, some rare☒ Magnetron sputtering ☒, and High‐entropy alloys☒	2.5	0.5	2
[78]	CC	In situ growth of Cu_3_P nanowires on Cu CC for uniform Na deposition	200 (pouch)	0.5C	In‐situ growth + Only Cu and P (low‐cost) ☑ Multi‐step synthesis ☒	4	3.5	4
[28]	CC	Use of a deformable Li metal layer beneath the Cu to improve Na plating uniformity and reduce SEI formation	100 (Coin)	C/3	Li‐Metal addition Contradicts Na‐Metal goal Multilayer☒	1.5	0.5	2
[83]	CC	Dual‐ion storage strategy with solvation‐free anion chemistry and CC engineering	500 (Coin)	2C	Plasma treatment (industrially used) ☑ Carbon coating ☑ Dual‐ion chemistry less mature☒	3.5	4	3
[108]	CC	Artificial sodium formate (HCOONa) interface on Cu CC	400 (Coin)	0.5C	Manual Na handling in the glovebox☒ Organic solvents, surfactants☒	3	0.5	2
[109]	CC	Sn‐monodispersed N‐doped mesoporous carbon nanofibers (SnNCNFs) as a 3D host for Na deposition	80 (Coin)	2C	MOF synthesis + hydrothermal + high‐temperature treatment☒ Low‐yield processes☒	1.5	1	2
[50]	CC	NST (Na_2_(Sb_2/6_Te_3/6_Vac_1/6_)) intermetallic skeleton as a sodiophilic CC	100 (Coin)	1C	Manual rolling/folding in the glovebox ☒ Antimony telluride (expensive, toxic) ☒	1	1	2
[82]	CC	3D porous carbon particles with carbon‐shell‐coated Fe nanoparticles (PC‐CFe) as a sodiophilic host for uniform Na deposition	100 (Coin)	1C	SPG membrane emulsification (specialized equipment) ☒ Block copolymer synthesis + dual crosslinking + carbonization at 900°C + Fe deposition + reduction at 500°C☒	1	0.5	2
[79]	CC	3D nanofiber‐like Zn coating on Al foil (Zn@Al) to enhance sodiophilicity and uniform Na deposition	100 (Coin)	0.5C	Magnetron sputtering ☒ Small batch processing ☒ Zinc target (low cost) ☑	0.5	1	2
[110]	CC	Zn foil as a sodiophilic CC for reversible Na plating/stripping	100 (Coin)	0.5C	Commercial Zn foil (cheap, abundant) ☑ Zero processing required☑ Direct industrial adoption possible☑	0.5	4.5	2
[111]	CC	Bulk‐integrated Na_2_Te/Zn/Mn framework enabling ultra‐low‐excess Na and 100% DOD operation	>300 (Coin)	0.5C	Multistep synthesis (solvothermal + annealing) with mechanical rolling/folding; partially scalable but air‐sensitive processing	2.5	2.5	3
[73]	CC	Fluorine‐integrated monolithic CNT current collector enabling AF pouch cells	200 (Pouch)	0.5C	One‐step solvothermal fluorination on CVD‐grown CNT scaffold; binder‐free, foil‐free architecture; compatible with scalable processing	4	4	4
[44]	CC	FCTF as a dual‐functional interphase (sodiophilic yet low Na^+^ depletion)	400 (Coin)	2C	Mild synthesis ☑ Pouch cell demonstration at low N/P ☑ Specialized monomers ☒ Complex organic synthesis ☒	3	3	3
[65]	CC + Elec.	Annealing and fluorination of Al for (100) ‐oriented F‐rich surface, enhancing Na nucleation and SEI stability.	50 (Coin)	0.3C	Simple HF treatment ☑ High Temperature operation☑ Al substrate ☑ Ionic liquid electrolyte (expensive) ☒	0.5	2.5	2
[21]	CC+ Elec.	Plasma, N‐doped carbon‐coated Al CC (p‐Al@C) + weakly solvated dual‐salt electrolyte (NaOTF/NaBF_4_ in G2) for cryogenic operation.	50 (Ah‐pouch)	0.05C	Air plasma treatment (industrially feasible) ☑ Carbon‐coated Al (scalable) ☑ Dual‐salt electrolyte (moderate cost) ☑	3	4.5	5
[55]	CC+ Elec.	Graphitic carbon coating, BPG electrolyte	260 (cylindrical)	C/6	Cylindrical cell ☑ GC@C ☑ Standard salts—commercial availability ☑ Ah‐level—relevant scale ☑	4.5	4.5	5
[48]	Elec.	Electrolyte engineering with fluorinated diluent (MPE) to form dense NaF‐rich SEI via dipole‐dipole interaction	700 (1.2 Ah pouch)	1C	Highly fluorinated electrolyte—MPE is extremely expensive (≈$500‐1000/kg) ☒ Specialized solvent system☒	5	2	3
[22]	Elec.	WSAS electrolyte (NaPF_6_ in 1,2‐diethoxyethane, DEE). Balances Na^+^‐anion interactions to form CIPs/AGGs clusters for stable SEI/CEI.	50 (Pouch)	0.1C/0.2C	Industrial pouch cells☑ Low Temperature operation ☑ DEE solvent—moderate cost ☑ High salt concentration ☒	3	3.5	3
[40]	Elec.	Sole‐solvent high‐entropy electrolyte (D2: diethylene glycol dibutyl ether + NaPF_6_/NaBF_4_). entropy‐driven salt dissociation, for stable SEI/CEI.	100 (Pouch)	1C	Multi‐layer pouch cells ☑ standard, commercial salts ☑ D2 solvent is more expensive than carbonates, but reasonable ☒	3.5	3	4
[23]	Elec.	3A zeolite molecular sieve film to create a highly aggregated solvation structure (AGG‐dominated) for enhanced oxidative stability (up to 4.5 V) and Na reversibility.	250 (Coin)	0.3C	Zeolite molecular sieve film—complex ceramic processing ☒ Precise pore size control (3.2Å) + manufacturing challenge☒	2	1.5	2
[71]	Elec.	Nano‐silica modified suspension electrolyte to suppress dendrites and enhance Na^+^ transport	50 (Coin)	1C	Simple additive approach + easy implementation ☑ Nano‐SiO_2_ – cheap, abundant material☑ Minimal process changes☑	1.5	4	3
[70]	Elec.	Electrolyte association‐dissociation equilibrium regulation using NaBF_4_ in hybrid G2/G4, to enhance desolvation and form inorganic‐rich SEI/CEI	100 (Pouch)	0.1C	Low‐cost salt☑ Simple electrolyte formulation.☑ Diglyme/tetraglyme solvents are more expensive than carbonates☒	3	2.5	4
[93]	CC+ SSE	Ferroelectric BaTiO_3_/C composite scaffold on Al CC to regulate Na^+^ flux via built‐in electric field	300 (Coin)	0.1C	Ferroelectric—complex ceramic processing☒ high‐temperature sintering—brittle ☒ Composite scaffold—multiple material integration☒	2.5	2	2
[30]	CC+ SSE	Electrochemically stable sodium borohydride solid electrolyte. Application of stack pressure to enable dense Na metal deposition. Pelletized Al CC to achieve intimate solid‐solid contact	400 (Coin)	0.8C	NaCrO_2_ cathode—low cost☑ Sodium borohydride ‐lower cost☑ Solid‐state processing—pelletizing, pressure requirements ☒ Multiple solid electrolytes—complex cell assembly☒	3	2	2
[94]	Separ.	Use of an in‐plane isotropic polyethylene (PE) separator to enable uniform Na plating and suppress short circuits.	100 (Coin)	1C	PE separators are commercially available and scalable. ☑ Al_2_O_3_ coating—established process☑	1	4.5	2
[87]	Cathode	Sodium‐free anode (SFA) configuration using presodiated Na_5_V_2_(PO_4_)_3_cathode as Na source.	300 (Coin)	2C	Established cathode material ☑ Presodiation step—adds manufacturing complexity/cost ☒ Not truly AF—defeats the cost/simplicity advantage ☒ Coin cell—but the process could potentially scale ☑	3.5	2.5	3

### 5.3 Pouch Cell Performance

The transition from coin to pouch cells is the decisive test of viability of AFSMBs. Pouch cell formats introduce practical constraints related to packaging, stack pressure, and electrolyte inventory, which reveal failure modes that remain concealed in flooded, small‐area coin cells [112]. A review of recent studies indicates that less than 10% of AFSMB reports include data on pouch cells, and only a subset of these reports meet industrially relevant metrics in terms of energy density, areal loading, and E/C ratio. Current advancements are concentrated in two areas: high‐energy‐density AF cells with moderate cycle life and long‐cycle anode‐less cells that compromise energy density (Figure 12c).

In the domain of high‐energy density, several studies have approached or surpassed the 200 Wh kg^−1^ cell‐level target under lean electrolyte conditions. An interfacial‐engineering approach, which combines a GC@Al collector with a BPG‐electrolyte, has achieved Ah‐level AF pouch cells at 205 Wh kg^−1^, with a layered‐oxide loading of over 15 mg cm^−2^ and an E/C ratio of 2 g Ah^−1^; these cells maintained their capacity for 260 cycles without applied stack pressure [55]. A functionalized porous aluminum current collector further enhanced the energy density: a 4.3 V AF‐pouch cell utilizing a layered oxide cathode achieved 420.4 Wh kg^−1^ (cathode‐ and anode‐based) with 84.9% retention after 100 cycles [107]. Sole‐solvent high‐entropy electrolytes extended performance across a broad temperature range: multilayer AF pouches cycled between ‐20 and 60°C, delivering 209 Wh kg^−1^ at the cell level with 83.1% capacity retention over 100 cycles and no gassing at 25°C [40]. Electrolyte‐equilibrium design has produced Ah‐level AF pouches that sustain 236, 231, 225, and 204 Wh kg^−1^ at 25, 0, −20, and −40°C, respectively, at 4.3 V [70]. Reported energy densities span approximately 200–210 Wh kg^−^^1^ at cell level and up to 420 Wh kg^−^^1^ on a cathode‐plus‐anode basis, although cycle life is generally limited to ≤100–260 cycles under lean E/C conditions.

Anode‐side reversibility is a necessary but not sufficient condition for high‐energy AFSMBs. Cell‐level specific energy is set jointly by cathode areal loading, operating voltage window, and cathode‐electrolyte compatibility, so improvements in plating efficiency do not translate proportionally unless the cathode supplies sufficient sodium at sufficient loading. This coupling is visible in the reported values compiled in Table 2: cells achieving the highest Coulombic efficiencies are frequently those operating at low cathode loading, where the anode‐side burden is smallest. Extending the voltage window raises specific energy but accelerates electrolyte oxidation and transition‐metal dissolution, the products of which migrate to the sodium surface and consume inventory (Section 3), so cathode and anode optimization are not separable. Reported energy densities should therefore be read together with their calculation basis, as cathode‐mass and active‐material values exceed cell‐level values by up to a factor of two.

The long‐cycle frontier is predominantly characterized by anode‐less or reservoir‐type designs. Hard carbon facilitated the development of an NFPP||Al‐HC pouch cell with a capacity of 2.28 Ah, an energy density of 203 Wh kg^−1^, an E/C ratio of approximately 2 g Ah^−1^, and a mere 0.026% capacity loss per cycle, equating to approximately 95% retention over extended cycling [46]. The utilization of FCTF as an interphase resulted in anode‐less pouches with an energy density of 325 Wh kg^−1^ (cathode+anode‐based) and robust stability over 300 cycles, while also enhancing the plateau‐to‐slope capacity ratio [44]. FMCNF supported an AF‐pouch with a high‐loading cathode that retained approximately 90% capacity over 200 cycles [66]. 3D N, P‐codoped carbon macroporous fibers embedded with CoP nanoparticles enabled a foldable NVP‐based AF‐pouch cell with stable cycling and approximately 67% capacity retention in long‐term tests [80]. Alloy‐modified collectors, such as Cu_3_P nanowires grown on Cu and In‐coated Cu foils, further extended stability: NVP||Cu_3_P@Cu pouches retained usable capacity for approximately 170 cycles from an initial 29 mAh at 0.5 C, while NFPP||Cu@In pouches demonstrated 78.3% retention after 320 cycles at 2 C with a loading of approximately 18 mg cm^−2^ [42, 78]. The trade‐off is direct: even a modest sodium reservoir or highly sodiophilic three‐dimensional host significantly enhances cycling robustness but reduces the maximum energy density compared to strict N/p = 0 designs.

Across these studies, a consistent “pouch cell penalty” is observed. Electrolytes and interfaces that achieve >99.9% CE in coin cells often decrease to approximately 99.0‐99.5% when applied to multilayer pouches at an E/C ratio of ≤3–4 g Ah^−1^. This penalty arises from several scale‐dependent factors: uneven stack‐pressure distribution, sodium plating at edges and tabs, larger effective areas for current‐collector corrosion, and the amplified impact of even small sodium losses when the electrolyte volume is limited. For instance, WSAS electrolytes that support long‐lived Cu||NFM coin cells exhibit marked capacity fading after only approximately 50 cycles in industrial multi‐layer pouches, despite maintaining a 74.3% capacity at −30°C [22]. Fluorinated‐carbon hosts that cycle >5000 times in symmetric or half‐cell tests show only approximately 200 stable full‐cell pouch cycles at high loading [66]. Furthermore, pouch formats introduce failure modes that do not appear in coin cells. Lean electrolytes magnify gas evolution from NaPF_6_ hydrolysis and solvent decomposition [113], leading to pouch swelling and delamination when E/C approaches 2–3 g Ah^−1^ [55, 70]. In true AF‐cells, every SEI repair event consumes active sodium; at 99.7% CE, a 3 mAh cm^−2^ cell loses 0.9 mAh cm^−2^ per 100 cycles solely to irreversible inventory loss [114], rapidly depleting the sodium inventory. Thin aluminum tabs and large‐area collectors create new corrosion pathways, enabling edge plating and internal shorts that are absent in coin cells with oversized tabs [42, 78]. These scale‐dependent phenomena explain why many “stable” coin‐cell chemistries fail when translated into practical pouches.

To facilitate cross‐study comparisons, it is imperative to establish stringent experimental standards for pouch cell research. Essential requirements include: (i) pressure‐controlled fixtures with in‐situ mapping to quantify stack‐pressure distribution; (ii) lean electrolyte protocols with E/C ≤ 3 g Ah^−1^ and humidity‐controlled filling (<1 ppm H_2_O) for pouch tests; (iii) systematic gas‐evolution measurement using mass spectrometry or pressure sensors; and (iv) post‐mortem Na‐inventory accounting via titration, cross‐sectional SEM, and ultrasonic mapping of delamination. Future research should focus on developing accelerated aging protocols that correlate short‐term pouch testing with long‐term durability projections and integrate pressure‐tolerant separators to mitigate compression failure. Pouch cells do not represent a simple geometric scaling of coin cells, but rather operate in a fundamentally different regime governed by coupled electrochemical‐mechanical constraints. Emerging pouch‐level AFSMB data indicate that cell‐level energies of 200–420 Wh kg^−1^ are attainable; however, long‐term stability at industrial areal capacities and lean E/C remains unresolved. Bridging this gap, therefore, requires not only improved materials but the co‐design of cell architecture, operating conditions, and manufacturing protocols.

## 6 Advanced Diagnostics and Predictive Design

Zero‐excess Na and fragile interfaces demand diagnostics that resolve chemistry and morphology in operando. Beyond electrochemical measurements, in situ and operando techniques have elucidated the causal relationships between interfacial chemistry, morphology, and failure (Figure 13a). Crucially, each diagnostic modality resolves distinct stages of the AF failure cascade, from nucleation heterogeneity and dead‐sodium formation to interphase evolution and cathode–anode crosstalk, enabling a direct link between observed phenomena and underlying degradation mechanisms.

**FIGURE 13:**
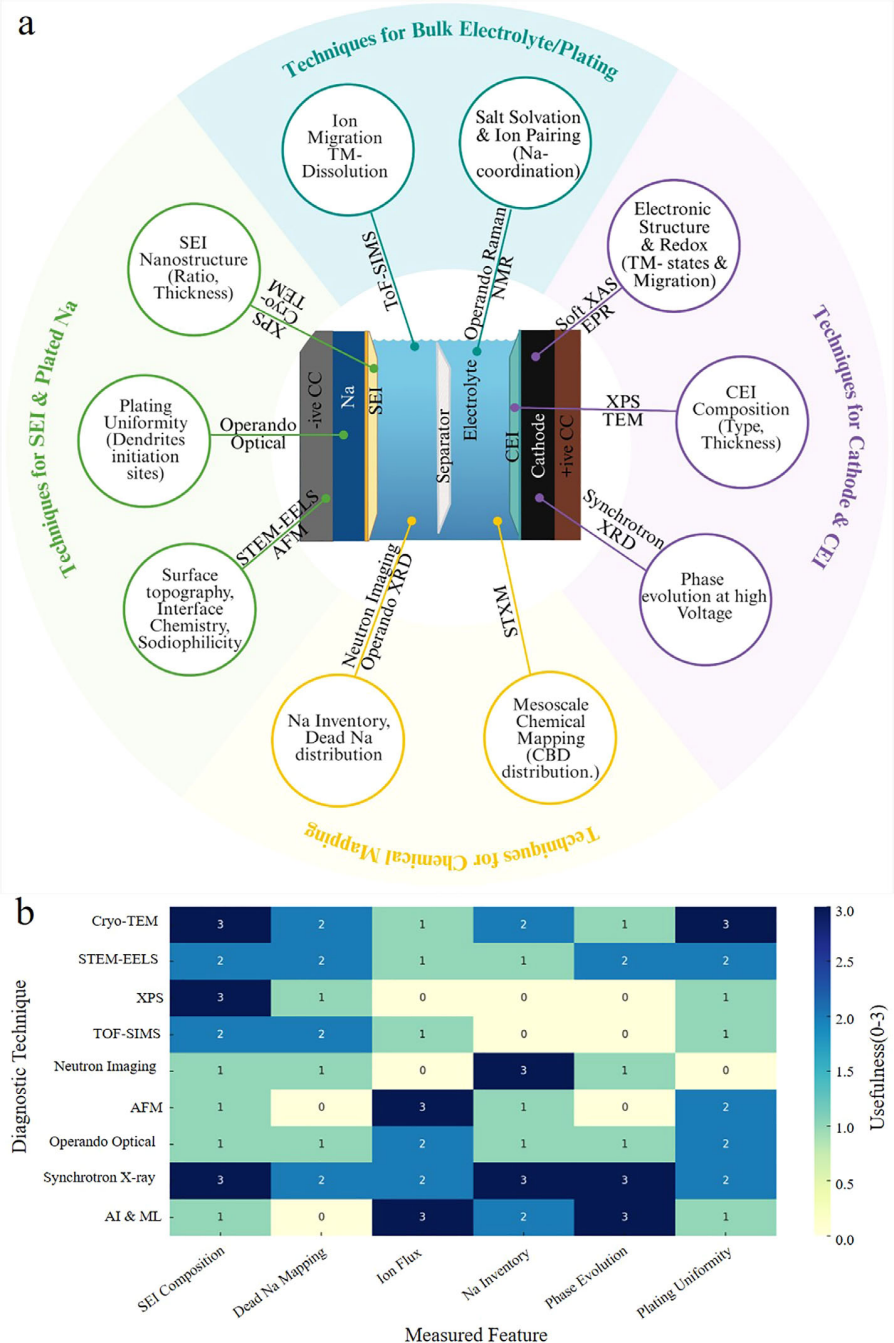
Mapping characterization techniques to failure modes and their effectiveness. (a) Spatial mapping of characterization techniques to failure modes in AFSMBs. A dissected AFSMB cell illustrates how specific analytical techniques probe distinct components and interfaces to reveal critical failure mechanisms, including SEI evolution, dead sodium formation, and cathode degradation. (b) Capability matrix for diagnostic techniques in AFSMB analysis. Quantitative assessment (0–3 scale) of technique effectiveness for probing key failure features, providing a strategic guide for experimental design, and highlighting Cryo‐TEM and synchrotron X‐ray as particularly versatile methods. The score matrix quantifies capability, while Table 5 states the limiting artifacts.

### 6.1 Nanoscale Interphase and Deposition Analysis

Direct visualization of sodium deposition is crucial for understanding AF behavior. Over the past three years, cryogenic and in situ transmission electron microscopy (TEM) has facilitated the nanoscale observation of fragile SEIs, dead‐sodium clusters, and nucleation pathways. Cryo‐TEM preserves native structures and has clarified SEI evolution under different additives. Cryo‐TEM has shown that in the absence of FEC, the SEI is disordered and organic‐rich, whereas the addition of 5% FEC resulted in a bilayered Na_3_PO_4_/NaF structure that supported stable cycling up to 800 h, compared to only 50 cycles at 0.5 mA cm^−2^ in control cells [54]. In AFSMBs, cryo‐TEM combined with electron energy loss spectroscopy (EELS) has been used to map chemical gradients and identify dead‐sodium hotspots (plasmon peak ≈ 3.1 eV) and Mn/Ni/Fe migration 10–20 µm into the SEI [22, 40, 55]. While cryo‐TEM offers static images, in situ TEM captures the dynamic processes of sodium plating and stripping. In situ TEM visualized the movement of Na nanocrystals along lattice planes, confirming morphology‐dependent deposition [115]. In situ TEM reveals that alkali metals exhibit Coble‐creep‐like viscous flow along interfaces under moderate pressure, facilitating void self‐healing and maintaining contact [103]. These observations indicate that metallic Na deforms plastically under moderate pressure rather than failing in a purely brittle manner [104].

These techniques are not interchangeable, and their complementarity is best expressed in terms of spatial resolution, chemical specificity, operando capability, and susceptibility to preparation artifacts (Table 5). Sodium is particularly demanding in this respect: it is soft, reactive toward trace moisture and oxygen, and has a low melting point (97.8°C), so that transfer, sectioning and beam exposure can each alter the state being measured [116]. Reported artifact sources include electron‐beam damage to interphase phases during microscopy, which constrains usable dose [117]; chemical reduction and preferential sputtering of interphase species during Ar^+^ depth profiling in XPS, which can bias the apparent inorganic fraction [118]; matrix effects in ToF‐SIMS that prevent conversion of secondary‐ion intensities into absolute concentrations [119]; and Ga^+^‐induced damage and local heating during FIB sectioning of metallic sodium [120]. Reported compositions should therefore be interpreted together with the preparation and transfer protocol used to obtain them. These limitations are directly relevant to closing the inventory balance of Equation (1). No single technique resolves all three terms: microscopy resolves morphology and the spatial distribution of residual sodium but samples volumes far smaller than an electrode; ^23^Na NMR distinguishes metallic from diamagnetic sodium quantitatively over the whole sample but without spatial resolution; and titration and ICP‐OES provide quantitative totals but are destructive and chemically unresolved. Determining *Q*_dead_ and *Q*_chem_ separately therefore requires the combination of a bulk‐quantitative measurement of residual Na^0^ with the electrochemically measured stripping capacity, supported by imaging to establish whether the residual metal is isolated or encapsulated. Adopting such combined protocols, rather than reporting composition or morphology in isolation, would allow the dominant loss channel to be identified rather than inferred.

**TABLE 5:** Complementarity and limitations of principal AFSMB diagnostics. Techniques compared across spatial resolution, chemical information, operando capability, and principal reported artifacts, with the sodium inventory term (Equation 1) each addresses. Complementary to Figure 9a, which scores capability, this table states the limiting artifacts. Artifact evidence marked as reported primarily for lithium systems is indicated as such.

Technique	Spatial resolution	Chemical information	Operando capability	Principal reported artifacts	Inventory term addressed
Cryo‐TEM (± EELS)	Atomic to nanometre	Phase and bonding, local	Limited (cryo, ex situ)	Beam damage above dose threshold; very small sampled volume	Q_dead_ (morphology)
In situ / operando TEM	Nanometre	Limited	Yes	Non‐representative cell geometry; beam‐induced effects	Q_dead_ (dynamics)
XPS (depth‐profiled)	∼10 µm lateral, nm depth	Quantitative, element and oxidation state	No	Ar^+^‐induced reduction and preferential sputtering; air exposure on transfer	Q_chem_
ToF‐SIMS	Sub‐µm lateral, nm depth	High sensitivity, molecular fragments	No	Matrix effects preclude absolute quantification; sputter‐induced mixing	Q_chem_
^23^Na NMR	None (bulk); mm with imaging	Metallic vs diamagnetic Na, quantitative	Yes (in situ)	Skin‐depth effects in metals; limited sensitivity to thin interphases	Q_dead_ + Q_stripped_
FIB‐SEM	Nanometre, 3D	Morphology only	No	Ga^+^ damage and heating of soft, low‐melting Na	Q_dead_ (volume, porosity)
Titration / ICP‐OES	None	Total elemental or Na^23^ content	No	Destructive; no spatial or speciation information	Q_dead_, Q_chem_ (totals)
Synchrotron X‐ray imaging	Sub‐µm, 3D	Limited for light elements	Yes	Beam‐induced heating; low contrast between Na and interphase	Q_dead_ (volume)

### 6.2 Operando and Mesoscale Observation

X‐ray photoelectron spectroscopy (XPS) and time‐of‐flight secondary‐ion mass spectrometry (ToF‐SIMS) are crucial for identifying SEI and CEI compositions that dictate reversibility. Depth‐profiled XPS tracks the evolution between inorganic NaF (688.5 eV) and organic C–O (286.2 eV) species, while several AFSMB studies have revealed CEI‐to‐SEI crossover reaction: BF_4_^−^‐derived fragments appear on both electrodes after 50 cycles, explaining the capacity fade [21, 55, 66, 70]. EELS, particularly when coupled with scanning TEM, complements these bulk probes by resolving nanometer‐scale gradients. Although still rare in AFSMBs, analogous analyses in Na‐ion and Li‐metal systems have distinguished inorganic SEI cores (LiF, NaF) from organic shells with sub‐10 nm precision [121, 122], highlighting the future relevance of this technique once Na‐sample stability improves.

Optical and scanning probe methodologies facilitate mesoscale observations. Operando AFM enables direct visualization of surface roughening and dendrite evolution, correlating these morphological changes with the voltage hysteresis [126]. Integrated cryo‐TEM, XPS, and in situ XRD approaches with a BeO‐window cell enable tracking of reversible phase transitions in Na_3_V_2_(PO_4_)_3_ and metallic Na without background interference [66]. The BeO peak served as a stable internal reference for quantifying the lattice shifts (Figure 9a).

### 6.3 Bulk and 3D Diagnostics

At larger length scales, neutron imaging and synchrotron‐based X‐ray techniques provide non‐destructive, bulk‐level diagnostics. Neutron tomography has quantified plating uniformity, improving from ±35% to ±8% with 3D collectors [127]. Synchrotron diffraction has mapped stress development in Na_3_Zr_2_Si_2_PO_12_ electrolytes, revealing crack initiation above approximately 2 MPa tensile stress [128]. Although beamline access limits widespread application, these methods uniquely visualize buried interfaces and Na inventory loss. A recent neutron diffraction study further demonstrated their capability to quantify in situ densification in solid‐state and hybrid architectures [129].

The collective impact of these complementary probes, as summarized in Figure 14, becomes most evident when viewed through their chronological progression. As illustrated in Figure 15a, diagnostic advances since 2019 delineate a clear trajectory from the early atomic‐scale imaging of native SEI layers to the current integration of multimodal datasets and machine‐learning‐assisted interpretation. This evolution has transformed AFSMB research from post‐mortem observations to proactive operando analyses. The expanding capability to interrogate degradation pathways in real time underpins the rational design of materials, interfaces, and full‐cell architectures.

**FIGURE 14:**
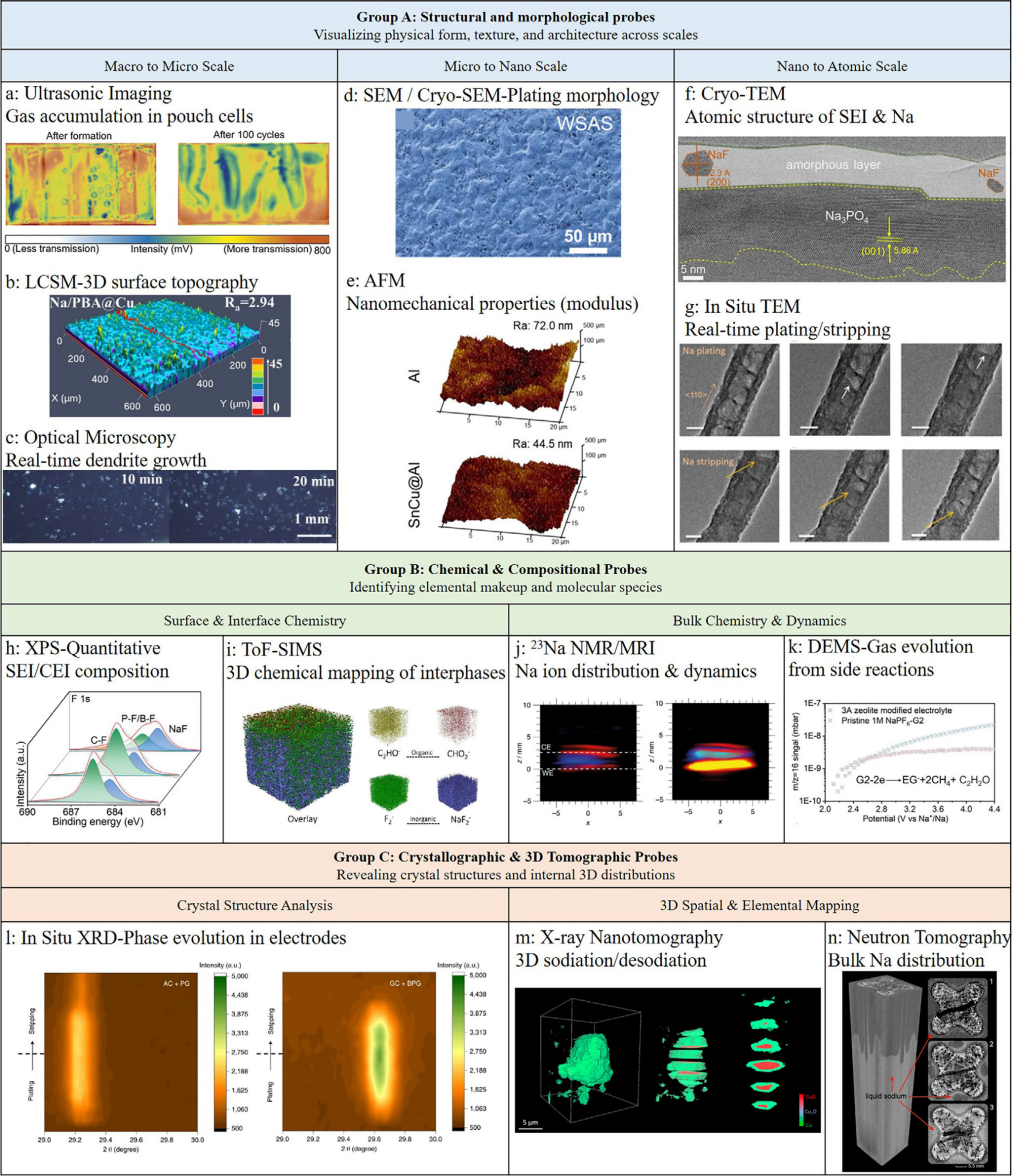
Overview of advanced characterization techniques for sodium‐metal batteries. (a–g) Structural and morphological probes visualizing physical form, texture, and architecture across scales. (h–k) Chemical and compositional probes identifying elemental and molecular species. (l–n) Crystallographic and 3D tomographic probes revealing crystal structures and internal spatial distributions. Panels (a, h) adapted with permission from [40]. Copyright 2025, Wiley. (b, c) Adapted with permission from [41]. Copyright 2025, American Chemical Society. (d) Adapted with permission from [22]. Copyright 2024, Wiley. (e) Adapted with permission from [84]. Copyright 2025, American Chemical Society. (f) Adapted with permission from [54]. Copyright 2021, (g) Adapted with permission from [103]. Copyright 2020 (i) Adapted with permission from [48]. Copyright 2025, (j) Adapted with permission from [123]. Copyright 2020, (l) Adapted with permission from [55]. Copyright 2022, Springer Nature. (k) Adapted with permission from [23]. Copyright 2022, Wiley. (m) Adapted with permission from [124], American Chemical Society. (n) Adapted with permission from [125], IOP Science.

**FIGURE 15:**
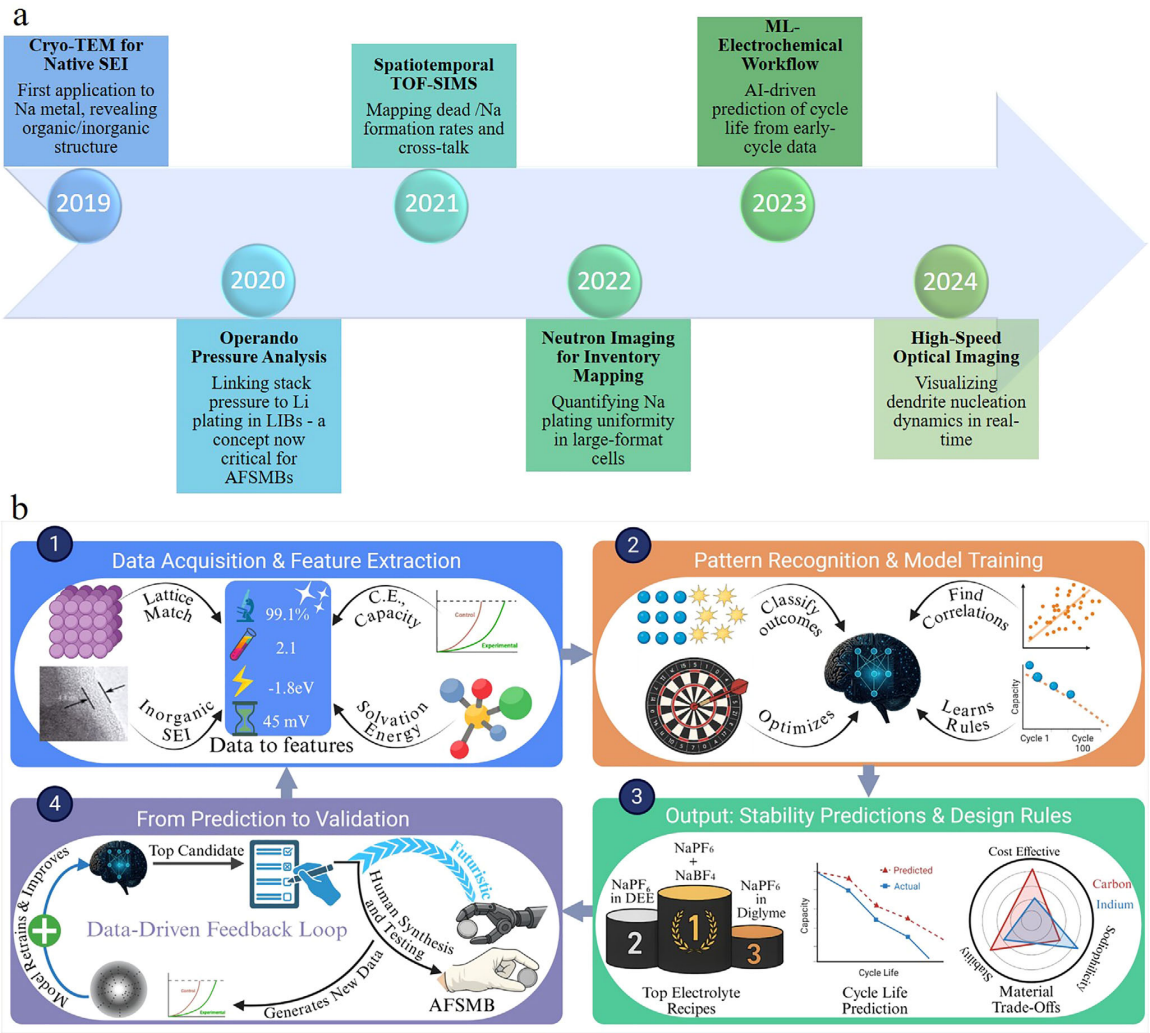
Operando diagnostic breakthroughs and machine learning‐accelerated discovery of AFSMBs. (a) Timeline of operando diagnostic breakthroughs in AFSMB research (2019‐2024). Evolution of characterization capabilities from initial interface analysis to sophisticated multimodal and machine learning approaches, marking pivotal advancements that have progressively decoded degradation mechanisms. (b) Machine learning workflow for accelerated AFSMB development. Integrated pipeline showing how multimodal data feeds into ML models for pattern recognition, prediction of cell performance, and autonomous optimization of materials and protocols, representing the next frontier in battery research.

### 6.4 Machine Learning and Modeling

While experimental diagnostics reveal degradation pathways, computational modeling and machine learning (ML) provide predictive and quantitative frameworks to interpret and control these processes. As AFSMBs are near practical deployment, understanding the intertwined morphological and interfacial dynamics is crucial. Computational modeling and ML offer complementary approaches to achieve this understanding. Phase‐field modeling, based on thermodynamics and kinetics, effectively simulates Na metal plating and stripping. These models quantify the influence of nucleation overpotential, ion flux gradients, and interfacial curvature on dendrite evolution and SEI growth. Recent Na‐specific adaptations that incorporate SEI diffusivity, electrolyte transport asymmetry, and Na‐SEI interfacial chemistry closely replicate operando observations. They demonstrated that high interphase stiffness and rapid Na^+^ transport within the SEI suppress protrusions, whereas surface roughness and concentration gradients induce early instability.

ML provides an alternative pathway for rapid screening and pattern recognition across compositional and structural spaces of materials. Recent studies have predicted electrolyte conductivity, classified degradation modes, and optimized interfacial architectures using limited data. Si et al. demonstrated accurate forecasting of Na^+^ conductivity using molecular descriptors and early cycle voltage data [130]. Models typically employ features such as the dielectric constant, solvation energy, porosity, crystallinity, and current density. Algorithms range from Bayesian optimization and support vector machines to deep convolutional or recurrent networks [131]. ML is particularly effective for amorphous interfacial materials, such as polymer SEI formers or nanostructured hosts, where deterministic models lack structural parameters, enabling high‐throughput linking of synthesis variables with ICE, long‐term CE, and state of charge [132]. ML‐driven image analysis now automates cryo‐TEM segmentation, enhancing the detection of dead sodium regions and dendrite propagation. This accelerates data interpretation and produces large annotated datasets for training. Such tools have advanced operando microscopy from correlative to causal understanding; for example, cryo‐TEM has been used to correlate inorganic‐rich SEI structures with improved electrochemical stability [133, 134].

Despite this progress, most models predict cycle life from electrochemical signals without resolving the interfacial processes that cause failure. This reflects the structure of the available data rather than a limitation of the algorithms. Voltage, capacity, and efficiency data are generated automatically, in large volumes and in a common format, and have supported accurate early‐cycle life prediction [44]; interfacial data are not. Sodium loss is reported almost exclusively as Coulombic efficiency, which aggregates the two loss channels of Equation (1) and therefore cannot serve as a mechanism‐specific label. Deposit morphology is likewise reported qualitatively, as mossy, porous, or dendritic, without the quantitative shape parameters, aspect ratio, packing density, porosity, and orientation, that would constitute a feature vector [52]. Testing conditions that govern these processes, including E/C ratio, stack pressure, areal capacity and cell format, are inconsistently recorded, so mechanism‐relevant variables remain confounded across aggregated datasets. Interpretability tools cannot recover what the features omit. Attribution methods such as SHAP and LIME rank the inputs supplied to a model [135]. Applying them to curve‐derived features therefore returns curve descriptors rather than interfacial mechanisms; their limited adoption is a symptom rather than the cause. Linking prediction to mechanism therefore requires mechanism‐level labels, specifically the separated inventory terms of Equation (1), quantitative morphological descriptors, and standardized reporting of operating conditions, in datasets large enough to train on. Hybrid frameworks that merge DFT‐derived Na‐binding energies with ML or use phase‐field outputs as synthetic training data can improve robustness and generalizability [136, 137].

Looking ahead, coupling ML with active learning and automated experimentation could create closed‐loop systems in which predictions guide experiments and new data continually refine the model (Figure 15b). For AFSMBs, where the material, interface, and architectural variables are tightly interdependent, such integrative modeling can replace empirical trial‐and‐error with rational design. The convergence of data‐driven intelligence and physical modeling is key to translating AFSMBs into stable and scalable energy‐storage technologies. Together, these advances shift AFSMB research from empirical observation to mechanism‐driven design, enabling the identification, quantification, and ultimately control of degradation pathways across multiple length scales. Furthermore, these diagnostic insights provide the foundation for identifying the remaining barriers to commercialization, discussed in the following section.

## 7 Future Perspectives and Commercialization

As AFSMBs transition from academic curiosity to industrial relevance, the field faces both technical uncertainties and commercialization challenges. The central challenge remains the debate over residual Na and the feasibility of fully reversible N/P = 0 operations. While theoretical models idealize complete reversibility, practical cells often retain 10–30% inactive Na trapped beneath fractured SEIs or within porous domains, undermining true AF cycling performance. Hybrid designs that incorporate trace Na reservoirs or sodiophilic interlayers, such as GC@Al with BPG electrolyte or p‐N@C@Al paired with dual‐salt NaOTF + NaBF_4_, have achieved > 99.9% CE under lean conditions while maintaining near‐zero N/P ratios [44, 52, 80, 138]. These results reflect meaningful progress toward reversible AF operation, although the acceptable threshold of residual Na for commercial classification remains unresolved. These challenges indicate that AFSMB commercialization is governed not by isolated material performance but by the integration of interfacial stability, sodium inventory retention, and manufacturing compatibility at the system level.

### 7.1 Industrialization Timeline

Strategic roadmaps have converged on phased development pathways spanning standardization, interfacial optimization, and architectural evolution. Although prototype demonstrations have reported Ah‐level pouch cells approaching 200 Wh kg^−1^, industrial validation remains limited, and most AFSMB strategies remain within TRL 2–4, with only a few reaching prototype‐level validation (TRL 5) (Table 4). Building on this assessment, Figure 16a systematically evaluates current AFSMB strategies through performance‐scalability mapping, revealing that only a minority of approaches simultaneously achieve high performance and scalability. To translate these system‐level constraints into actionable pathways, development priorities can be organized across short‐, mid‐, and long‐term horizons (Figure 16b). In the short term (2024–2026), the immediate priority lies in standardizing pouch‐cell testing protocols. Current inconsistencies in pressure application, E/C ratios, and N/P calibration obscure cross‐study comparisons and hinder industrial translation [139, 140]. Agencies such as the U.S. DOE, through the Battery500 and B‐2030 initiatives, have emphasized the need for testing under >3 mAh cm^−2^ loading, with CE >99.96% and electrolyte usage below 2 g Ah^−1^. Establishing these criteria across academic and industrial labs is a foundational step [4, 141, 142]. In the mid‐term (2026‐2028), the focus will shift to electrolyte‐SEI co‐design and current collector innovation. Strategies such as additive cocktails, high‐donor ether systems, and nanostructured or doped interphases (e.g., FCTF and p‐N@C@Al) will be key to achieving stable cycling under lean and ambient conditions. Simultaneously, industrially scalable architectures, such as electrospun fluorinated carbon frameworks or coated Al foils, must be validated at the Ah‐level. In the long term (2028‐2030), the development trajectory extends toward solid‐state AFSMBs for grid‐scale storage and mobility applications. The elimination of flammable liquid electrolytes improves safety and energy density while mitigating dendrite‐related challenges in conventional systems [30]. These directions are consistent with emerging industrial roadmaps targeting cost reductions below $50 kWh^−1^ and deployment in stationary and flexible energy systems [143, 144, 145].

**FIGURE 16:**
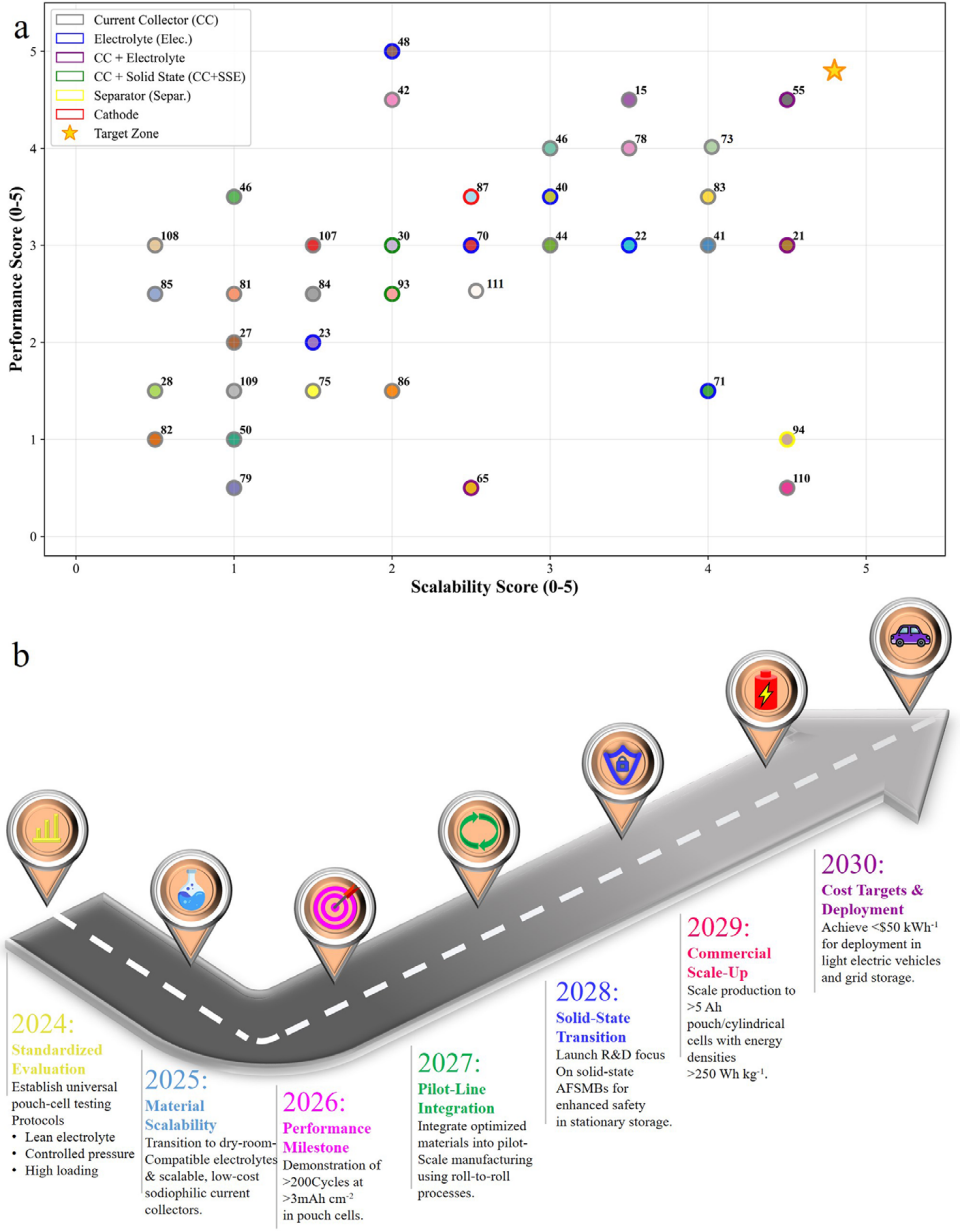
Technology readiness matrix and development roadmap for AFSMBs. (a) Systematic evaluation of AFSMB strategies plotted by performance versus scalability scores, using the scoring scheme summarized in Table 4. Bubble colors represent individual studies, while outlines indicate strategy categories. The gold star marks the target zone for commercially viable technologies. (b) Proposed technology roadmap outlining critical milestones from standardized laboratory evaluation (2024) to commercial deployment (2030), targeting key challenges in materials processing, cell integration, and manufacturability.

Despite promising laboratory results, the absence of consistent TRL 6–7 demonstrations highlights a fundamental disconnect between material innovation and manufacturable cell architectures. The industrialization of AFSMBs will require coordinated partnerships between material suppliers, electrolyte formulators, and battery manufacturers. While leading companies such as Faradion, HiNa, CATL, and Altris currently focus on Na‐ion architectures with hard‐carbon anodes, their manufacturing frameworks provide a natural stepping stone toward integrating AFSMB‐compatible chemistries. Emerging pilot‐scale demonstrations, including pouch cells with capacities of 1.2‐5 Ah and energy densities approaching 200 Wh kg^−1^, indicate early progress toward practical deployment. However, critical gaps persist: coin‐cell CE performance (>99.96%) often fails to translate to large‐format prototypes due to unoptimized stack pressure, inconsistent electrolyte dosing, and lack of dry‐room‐compatible assembly. TRL assessment reveals that most AFSMB strategies remain at early development stages (TRL 2–4), with only a few approaches advancing to prototype validation (TRL 5) (Table 4). The scarcity of TRL 5–6 demonstrations indicates the challenge of translating from the lab‐to‐fab. Strategies employing commercially compatible materials and processes (e.g., [60, 84, 108]) show higher TRL scores, while complex syntheses and rare materials consistently correlate with lower TRL (2–3). This TRL gap highlights the urgent need for coordinated efforts to advance promising concepts toward pilot‐scale validation.

### 7.2 Sustainability and Research Priorities

Material sustainability presents a compelling argument for AFSMBs over Li‐based systems. Na is more abundant than Li in the Earth's crust and is distributed across geopolitically diverse regions, including India, China, the U.S., and parts of Europe, mitigating the supply chain vulnerabilities currently faced by Li. Furthermore, the ability to use Al current collectors in AFSMBs, rather than Cu, reduces both cost and embodied energy [146, 147]. From a lifecycle perspective, Na‐based batteries exhibit 30–50% lower cradle‐to‐gate CO_2_ emissions compared to NMC‐based Li‐ion systems. The absence of critical transition metals such as cobalt and nickel, and the use of environmentally benign salts like NaPF_6_ and NaBF_4_, further aligns with green energy mandates. For applications in grid‐scale storage or sustainable mobility, these advantages position AFSMBs as an environmentally and economically favorable option [148, 149]. However, these advantages are contingent on replacing fluorinated salts and moisture‐sensitive electrolytes with scalable, environmentally compatible formulations.

Four scientific challenges, distinct from engineering scale‐up, remain unresolved. First, the sodium inventory is not routinely decomposed: reporting Coulombic efficiency alone aggregates morphologically and chemically driven losses that respond to different design levers, and until the terms of Equation (1) are measured separately, the dominant failure channel in a given system is inferred rather than determined. Second, the causal role of interphase composition is unestablished. The correlation between inorganic‐rich interphases and reversibility is reproducible, but ether systems achieving comparable efficiency from a different solvation regime indicate that composition is an incomplete descriptor, and discriminating experiments at matched conditions have not been performed. Third, calendar behavior is essentially uncharacterized. Plated sodium at high state of charge corrodes without cycling, and inventory loss during rest is not reported in any of the systems surveyed here, despite being decisive for a reservoir‐free architecture. Fourth, no quantitative morphological descriptor is in general use: deposits are classified qualitatively, which prevents both predictive modeling and the mechanism‐level labeling that machine‐learning approaches require. Addressing these does not require new materials so much as new measurement and reporting conventions, and progress on them would make the engineering challenges of pressure control, electrolyte leanness, and dry‐room processing tractable rather than merely acknowledged.

To catalyze the commercialization of AFSMBs, we propose a coordinated “Battery300” initiative, modeled after the DOE Battery500 program, to unify evaluation standards, accelerate cross‐sector collaboration, and align academic innovation with industrial requirements. This framework would bring together academia, national laboratories, and industry around key milestones: (i) Development of AFSMBs with target energy density ≥300 Wh kg^−1^; (ii) Achievement of CE >99.95% under Ah‐scale pouch cell conditions; (iii) Engineering of stable SEIs and CEIs for lean‐electrolyte, ambient‐environment operation; (iv) Definition of standardized metrics for truly AF (N/P = 0) systems; (v) Establishing open‐access protocols and shared design databases for benchmarking and validation. Such coordinated efforts would accelerate the transition from laboratory demonstrations to manufacturable technologies while addressing remaining challenges in supply chains, processing, and policy alignment. Ultimately, the future of AFSMBs lies not in maximizing isolated performance metrics, but in achieving integrated, manufacturable systems that couple interfacial precision with industrial scalability.

## 8 Conclusion

AFSMBs are not simply high‐energy systems without excess metal, but sodium‐inventory‐limited architectures in which performance is governed by nucleation behavior, interphase stability, cross‐electrode interactions, and manufacturability under realistic conditions. In practice, incomplete reversibility and the accumulation of dead sodium make it difficult to sustain a true N/P = 0 operation. Progress on this point is limited by measurement convention as much as by materials: because reported Coulombic efficiency aggregates morphologically and chemically driven losses, the dominant failure channel in a given system is usually inferred rather than determined. Although substantial progress has been achieved through interfacial and architectural design, a unifying limitation emerges: improvements in individual components rarely translate into stable, practical cells. Of twenty‐four representative systems surveyed here, only one satisfies both lean‐electrolyte and high‐areal‐capacity criteria simultaneously, indicating that AFSMBs are fundamentally integration‐limited rather than materials‐limited systems. Key mechanistic challenges remain unresolved, including the pathways of dead‐sodium accumulation, the coupled evolution of SEI and CEI under lean and pressure‐dependent conditions, and the role of cross‐electrode degradation in accelerating failure cascades. Addressing these challenges requires a shift from coin‐cell optimization to standardized evaluation protocols that capture initial Coulombic efficiency, long‐term reversibility, electrolyte consumption, cathode loading, and stack‐pressure effects under practical conditions. Future progress will depend on integrating operando diagnostics with modeling and machine learning to establish predictive design principles. More importantly, the development of AFSMBs must transition toward co‐optimized systems in which current collectors, electrolytes, cathodes, separators, and processing conditions are engineered collectively. Ultimately, the realization of AFSMBs will not be determined by isolated materials advances, but by the ability to translate interfacial precision into integrated, manufacturable cell architectures that operate reliably under industrially relevant conditions.

## Author Contributions

**Hamid Hussain**: writing – original draft, visualization, investigation, conceptualization. **Shazaib Ali**: writing – original draft, visualization, investigation, conceptualization. **Muhammad Ali**: writing – review and editing, visualization. **Samia Aman**: writing – review and editing, visualization. **Moazzam Ali**: writing – review and editing. **Ufra Naseer**: writing – review and editing. **Sajid Farooq**: writing – review and editing. **Sailing He**: writing – review and editing. **Asif Mahmood**: writing – review and editing. **Muhammad Yousaf**: writing – review and editing, supervision, funding acquisition, conceptualization. **Yinzhu Jiang**: writing – review and editing, supervision, funding acquisition, conceptualization.

## Conflicts of Interest

The authors declare no conflicts of interest.

## Data Availability

The data that support the findings of this study are available from the corresponding author upon reasonable request.
